# First Application of Artificial Neural Networks to Estimate 21st Century Greenland Ice Sheet Surface Melt

**DOI:** 10.1029/2021GL092449

**Published:** 2021-08-19

**Authors:** Raymond Sellevold, Miren Vizcaino

**Affiliations:** ^1^ Geoscience and Remote Sensing Delft University of Technology Delft The Netherlands

**Keywords:** Greenland ice sheet, surface melt, machine learning, neural networks

## Abstract

Future Greenland ice sheet (GrIS) melt projections are limited by the lack of explicit melt calculations within most global climate models and the high computational cost of dynamical downscaling with regional climate models (RCMs). Here, we train artificial neural networks (ANNs) to obtain relationships between quantities consistently available from global climate model simulations and annually integrated GrIS surface melt. To this end, we train the ANNs with model output from the Community Earth System Model 2.1 (CESM2), which features an interactive surface melt calculation based on a downscaled surface energy balance. We find that ANNs compare well with an independent CESM2 simulation and RCM simulations forced by a CMIP6 subset. The ANNs estimate a melt increase for 2,081–2,100 ranging from 414 ± 275 Gt ${yr}^{-1}$ (SSP1‐2.6) to 1,378 ± 555 Gt ${yr}^{-1}$ (SSP5‐8.5) for the full CMIP6 suite. The primary source of uncertainty throughout the 21st century is the spread of climate model sensitivity.

## Key Points

- We explore connections between atmospheric variables from global climate simulations and Greenland melt using neural networks
- The neural networks' melt predictions compare well to those calculated by a global and a regional climate model
- Climate model spread is the main source of uncertainty in 21st century Greenland melt projections

## 1 Introduction

Greenland is losing mass at an accelerating rate since the 1990s (Bamber et al., 2018; Mouginot et al., 2019; Shepherd et al., 2020) in response to global warming. This is alarming, as the freshwater stored on the Greenland ice sheet (GrIS) has the potential to raise the global mean sea level by 7.34 m if fully melted (Bamber et al., 2018). The current mass loss of the GrIS is the result of increased ice discharge and decreased surface mass balance (SMB), with the latter being the dominant contributor (Fettweis et al., 2017; van den Broeke et al., 2016), and the cause of mass loss acceleration (Enderlin et al., 2014; Shepherd et al., 2020). The main contributor to this contemporary GrIS surface mass loss is increased surface melt (Fettweis et al., 2017; Noël et al., 2020). Projections of future GrIS surface melt are scarce, as most global climate models do not feature a (realistic) melt calculation (Cullather et al., 2014; Lenaerts et al., 2019; Vizcaino et al., 2014).

The classical approach to use climate simulations for estimates of ice sheet surface melt is through positive‐degree‐day schemes (Braithwaite, 1995; Wake & Marshall, 2015). While these schemes are often used for computational efficiency, their performance is poor when surface melt/ablation is high (Bauer & Ganopolski, 2017), which we can expect for most global warming scenarios. More advanced, state‐of‐the‐art 21st century projections of GrIS surface melt come from RCMs (Fettweis et al., 2013; Mottram et al., 2017; van Angelen et al., 2013). These models are run at high resolution and with a surface energy balance‐based calculation of melt. However, they are computationally expensive and require external forcing from a global climate model (Noël et al., 2020).

Here, we investigate the performance of artificial neural networks (ANNs) in translating climate projections from the global models participating in the Climate Model Intercomparison Project Phase 6 (CMIP6; Eyring et al., 2016) into GrIS surface melt projections. ANNs are computationally efficient and able to learn complex nonlinear relationships. We explore whether ANNs can learn the relationship between quantities simulated by a global climate model and simulated melt through training with output from the Community Earth System Model version 2.1 (CESM2; Danabasoglu et al., 2020). CESM2 features an advanced, interactive calculation of the GrIS surface melt based on downscaling of the surface energy balance (Sellevold et al., 2019; van Kampenhout et al., 2017, 2020). This study aims to take the first steps toward further use of global climate models and ANNs to project GrIS surface melt.

## 2 Methods

### 2.1 Climate Data for Training

To train the neural networks, we use the model output from CESM2 (Danabasoglu et al., 2020), an advanced Earth system model with components for atmosphere, ocean, sea‐ice, land, and (optionally) land ice. It is run with a nominal 1° horizontal resolution. Further, it features a surface melt calculation in the land component with advanced snow physics (Flanner & Zender, 2006; van Kampenhout et al., 2017) and elevation classes downscaling (Sellevold et al., 2019). This calculation is evaluated in van Kampenhout et al. (2020) and applied to simulate future evolution of GrIS SMB in Sellevold and Vizcaíno (2020) and GrIS dynamical evolution in and Muntjewerf, Petrini, et al. (2020).

We use annual CESM2 melt rates from 10 historical and 19 scenario simulations (Table S2 and Figure S1). These CESM2 simulation years were randomly divided into two parts: the first to train the neural networks (90%), and the second for cross‐validation (that is, to tune the neural networks, 10%).

### 2.2 Artificial Neural Network

We designed the ANNs to predict annual melt by independently using summer (JJA) averages of five atmospheric variables: near‐surface temperature ($T_{2m}$), 500 hPa geopotential heights ($Z_{500}$), cloud cover (CC), incoming radiation (${RAD}_{in}$; the sum of incoming shortwave and longwave radiation), and snowfall (SNOW). These variables were chosen as they have been identified to be connected to melt increase (Hanna et al., 2016; Hofer et al., 2017; Tedesco & Fettweis, 2020a). After preliminary testing, we decided to train an ANN per variable versus a combination of variables to avoid the network focusing on one variable more than others, as individual variables may be biased in CESM2 or other climate models. Further, using individual variables for each ANN allows for uncertainty quantification based on variable selection.

Before feeding the climate data to the ANNs, we apply a preprocessing step. The data is scaled (Table S1) to the range 0.2–0.8 to make the network find the optimal combination of weights more efficiently. Further, the data is transformed from two‐dimensional maps to a one‐dimensional vector. The resulting vector is of size *n* = number of latitude points × number of longitude points = 192 × 288 = 55,296. The maps are global rather than cropped to a user‐defined region around Greenland, as we want the neural networks to determine their region(s) of interest.

The ANNs consist of an input layer, a hidden layer, and an output layer (schematic of architecture in Figure S2). The input layer is the first layer of the ANN and takes in the vectorized data. The input is then passed to each of the units in the hidden layer. The hidden layer consists of four units. This number of units was chosen after testing with different initialization methods and hyperparameters, where the number of units activated by the networks never exceeded four. Each unit (i) holds its unique weight vector $\vec{W_{i}}$ of same size as $\vec{X}$. These weights are randomly initialized (Glorot & Bengio, 2010). For each unit, we calculate a scalar $F_{i}$:

(1) $$F_{i}\left(\vec{W_{i}},\vec{X}\right)=B_{i}+\vec{W_{i}^{T}}\cdot \vec{X},$$

where $B_{i}$ is the bias. We then apply an activation function, which transforms $F_{i}$ to zero if $F_{i}\;<$ 0, and does nothing if $F_{i}\;\geq$ 0 (also known as rectified linear unit activation; Xu et al., 2015).

When optimizing these weights, we use simple ridge regression (also known as Tikhonov regularization with units regularization matrix or L2 regularization). Ridge regression applies a penalty (λ; Table S1) for large weights during optimization to ensure that weights do not grow too large. This is a common technique to avoid overfitting and to derive physically coherent patterns (Barnes et al., 2019). The weights of each unit can be restructured into the gridded format of the training data. Weights restructured in this way are referred to as feature maps.

The output layer gives the predicted melt rates as the annual integrated GrIS melt. It predicts the melt through

(2) $$MELT=b+\sum_{i=1}^{4}F_{i}\cdot w_{i}$$

where *b* is a bias term, and $w_{i}$ are the weights in the output layer. They are calculated as in the hidden layer, though, without the use of an activation function.

Training of the network is done through a feedforward and backpropagation algorithm. The data is sent through the network in batches of 30 samples in the feedforward. One sample is equal to one summer average of the input variable with its corresponding annual melt. The samples in each batch are selected randomly from the available training data, so the network does not learn the time order. The network generates a melt prediction, and the mean squared error between the predicted melt and the melt as simulated by CESM2 is calculated. To minimize the error, backpropagation and gradient descent are performed. Backpropagation computes the gradient of the loss function (mean squared error) with respect to the weights efficiently. We use the adaptive moment estimation (ADAM) gradient descent algorithm to optimize the ANNs' weights to minimize the loss function. ADAM is a stochastic algorithm with exponential decay of the learning rate (Kingma & Ba, 2014). Here, we use learning rates in the range of $10^{-3}$ – $10^{-4}$ (Table S1). We use 0.9 for the first moment exponential decay rate and 0.999 for the second raw moment exponential decay rate.

Once all available batches of samples are through the network, an epoch has ended. Before starting a new epoch, the samples are randomly shuffled and divided into new batches. We let the ANNs train for 20,000 epochs and save the ANNs' state where the melt predictions are the closest to the explicitly calculated melt for the cross‐validation data. The number of epochs needed was in the range of 630–19,757 (Table S1), depending on the variable.

### 2.3 Climate Data for Melt Projection

We use the output from all global climate models participating in the CMIP6 to project GrIS surface melt for the historical period, and four Shared Socioeconomic Pathways (SSPs; O'Neill et al., 2016) (Table S2). In total, we use 345, 123, 147, 147, and 129 simulations from 46, 30, 32, 28, and 32 climate models for the historical period, SSP1‐2.6, SSP2‐4.5, SSP3‐7.0, and SSP5‐8.5, respectively.

We bilinearly interpolate the global climate model output to the CESM2 ∼1° horizontal grid to be compatible with the ANNs' input layers. Further, we use climate anomalies with respect to the 1979–1998 mean climatology, which we add to the CESM2 1979–1998 ensemble‐mean climatology. This avoids potential artifacts in the projections due to a different baseline climatology than used to train the ANNs. Therefore, we only assess the melt increase due to a change in the climate forcing. We do not discard any simulations if some variables are not available.

## 3 Results

### 3.1 Analysis of the Neural Networks

**Figure 1:**
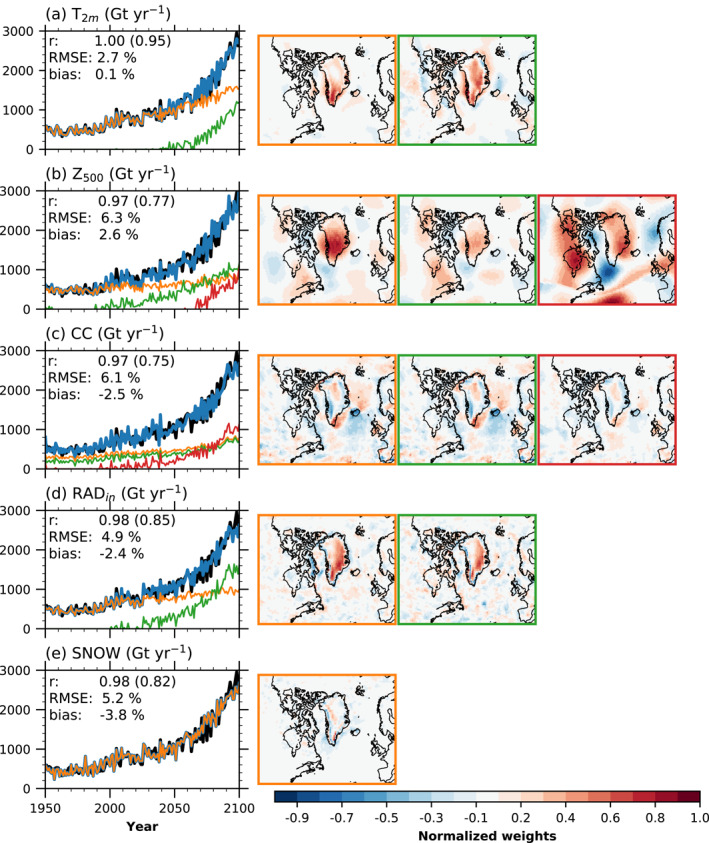
Comparison of artificial neural network (ANN) melt projection (blue line) from five (summer‐mean, JJA) climate variables with melt explicitly simulated (black line) for the same independent historical/SSP5‐8.5 CESM2 climate simulation (Noël et al., 2020): (a) from near‐surface temperature, (b) from 500 hPa geopotential heights, (c) from cloud cover, (d) from incoming radiation, and (e) from snowfall, all in Gt ${yr}^{-1}$. The orange, green, and red lines correspond to the melt contribution from the feature maps with matching frame color to the right of the time series. The sum of the colored lines equals the total ANN predicted melt (blue line). The annotated *r* shows the correlation coefficient between the explicitly calculated CESM2 melt and the ANN predictions, with the detrended (by subtracting 20 years running mean) correlation coefficient in parentheses. The root mean squared error (RMSE) for the entire time series and bias in the last 20 years of the ANN predictions are shown as a percentage of the CESM2 calculated melt in the last 20 years.

To demonstrate our method, we use the ANNs to predict melt from five atmospheric variables obtained from an independent CESM2 historical/SSP5‐8.5 simulation not used for training or cross‐validation and compare to the explicit melt calculation in this simulation. Figure 1 shows the feature maps corresponding to each activated function (the weights learned by the ANNs in the hidden layer; $\vec{W_{i}}$). Here, we only show weights in the Greenland region, as weights from the other areas are much smaller (Figure S3).

All the input variables have high predictive skill when compared with the explicit melt calculation in CESM2 (see annotations in Figure 1). The correlation coefficients show that they can both capture the melt increase and the interannual variability. Further, predictions with $T_{2m}$ and $Z_{500}$ overestimate melt, while predictions with CC, ${RAD}_{in}$ and SNOW underestimate melt.

An advantage of the ANNs is their ability to represent nonlinearities in the melt evolution, even if they are not present in the input data. This is achieved by applying activation functions to the hidden layer units. For instance, the function for $T_{2m}$ represented by the green line (and corresponding to the map with the green frame) activates around 2040 and sharply increases around the years 2060–2065 (Figure 1a). 2060–2065 corresponds to the timing of acceleration of simulated melt (year 2071, according to a breakpoint analysis; Muntjewerf, Sellevold, et al., 2020).

For $T_{2m}$, the highest weights in the first map (orange frame) are over the GrIS, particularly at the southern dome. Also, temperatures over the adjacent ocean are weighted, indicating a connection between warmer oceans near Greenland and increased melt. All these weights are positive, indicating that rising temperatures and increased melt are connected. The feature map representing the year 2060 acceleration (green frame) weighs the interior and over the oceans more strongly. This is due to the interior of the ice sheet warming more than the margins during summer with global warming (Fettweis et al., 2013). As the ice surface cannot exceed the melting point, the warming of the atmosphere directly above is to some extent limited (Vizcaino et al., 2014). Also, Arctic Ocean temperatures close to the northeastern part are positively weighted in connection with major summer sea‐ice reductions after 2060.

For $Z_{500}$ (green line), CC (red line), and ${RAD}_{in}$ (green line), the activation of an additional feature takes place in between 1990 and 2000 (Figures 1b–1d). Further, these activated features, or melt contributions, increase around the year 2020. These two timings correspond well to the melt increases simulated around these periods. The 2060–2065 melt increase coincides with the activation and increase of another unit for $Z_{500}$ (red line).

The connection between $Z_{500}$ and melt is well established (Box et al., 2012; Delhasse et al., 2018; Hanna et al., 2016; Sellevold & Vizcaíno, 2020). Increased $Z_{500}$ promotes more heat advection, clear skies (Hofer et al., 2017) and blocking. Also, negative $Z_{500}$ anomalies, indicative of more cyclones, are linked to melting by increasing rainfall over the GrIS (Oltmanns et al., 2019). The melt prediction strongly weights $Z_{500}$ over the GrIS (Figure 1b, orange frame). $Z_{500}$ is also negatively weighted over the Labrador Sea. For the post‐2000 activation (Figure 1b, green frame) similar, though less strong, patterns appear. A third activation happens around the year 2065, with the associated patterns in Figure 1b, red frame. In contrast to the previous feature maps, the center of the positive weights over the GrIS moves from central Greenland to the eastern coast and a weaker center in the northwest. Further, the negative weights over the Labrador Sea intensify.

The CC maps that are active from the beginning of the ANN prediction are very similar (Figure 1c, orange and green frames). They predict more melt if cloud cover decreases around the margins or more melt if cloud cover increases in the interior. This is likely due to the competing effects of increased incoming shortwave and decreased incoming longwave radiation with decreasing cloud cover, and vice versa (Izeboud et al., 2020; Wang et al., 2019). For high albedo surfaces, such as the snow‐covered interior, reduced cloud cover increases incoming shortwave radiation for a larger area. However, since a high amount of this radiation is reflected, this has minimal impact on the surface energy balance. If cloud cover increases, incoming longwave radiation increases, resulting in more energy at the surface. For a low albedo surface, such as the bare ice exposed margins, the effect is opposite, and thus decreasing the cloud cover increases the energy at the surface more than if cloud cover increases. From around the year 2000, a third feature map is activated (Figure 1c, red line and frame), which is also similar to the previously activated feature maps.

The two feature maps for ${RAD}_{in}$ are very similar (Figure 1d). They show that increasing ${RAD}_{in}$ over the GrIS surface increases the projected melt. Interestingly, the weights show that increased ${RAD}_{in}$ in the south—southeast is the strongest indicator for melt increase. A plausible explanation for the similar feature maps is that the evolution of incoming radiation is rather linear, so it cannot account for the nonlinear increase in the surface melt. So another feature needs to activate to account for the nonlinear increase in the melt, likely due to lowering the GrIS' surface albedo with elevated global warming.

The melt prediction based on SNOW (Figure 1e) shows that more melt is predicted with reduced SNOW at the margins and increased snow in the interior. Less summer snowfall at the margins is linked to higher melt rates (Noël et al., 2015; Tedesco & Fettweis, 2020b), by not temporarily increasing the albedo and interrupting bare ice exposure. For the interior, this pattern is likely aliasing of the evolution due to a warming world: increasing the global temperatures leads to more snowfall in the interior (Mottram et al., 2017).

One interpretation of the activation of additional features can be that the melt accelerates when they are activated (e.g., $T_{2m}$ and $Z_{500}$, Figures 1a and 1b). However, this also depends on the input variable. If the input variable follows a similar evolution as melt, the surface melt is predicted without activation of additional features (e.g., SNOW). So another interpretation of the activation features can be that they represent feedback mechanisms, such as the melt‐albedo feedback (Box et al., 2012), that accelerates melt as ablation areas expand (Muntjewerf, Sellevold, et al., 2020; Sellevold & Vizcaíno, 2020).

### 3.2 Comparison With Regional Climate Modeling

**Figure 2:**
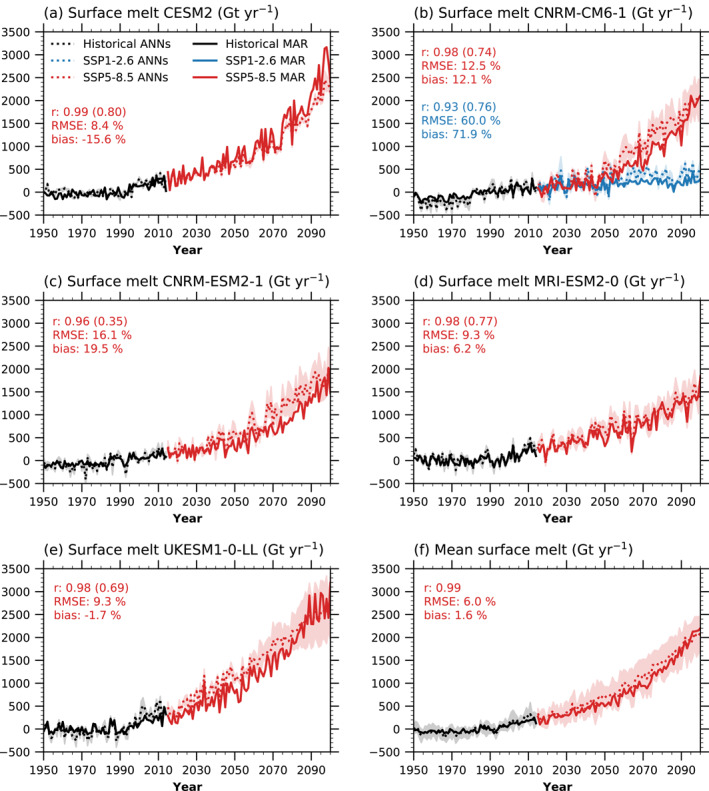
1950–2100 time series of Greenland ice sheet (GrIS) integrated surface melt change (Gt ${yr}^{-1}$), with respect to 1979–1998, in Modele Atmospherique Regional (MAR) and the artificial neural networks (ANNs) (mean of $T_{2m}$ and SNOW) for an individual climate simulation from (a) CNRM‐CM6‐1, (b) CNRM‐ESM2‐1, (c) UKESM1‐0‐LL, (d) MRI‐ESM2‐0, and (e) CESM2. Panel (f) shows the mean of (a–e). Black, blue, and red dotted (solid) lines correspond to the historical, SSP1‐2.6, and SSP5‐8.5, respectively, as projected by the ANNs (MAR). The annotated *r* shows the correlation coefficient between the explicitly calculated MAR melt and the ANN predictions, with the detrended (by subtracting 20 years running mean) correlation coefficient in parenthesis. The root mean squared error (RMSE) for the entire time series and bias in the last 20 years of the ANN predictions are shown as a percentage of the MAR simulated melt change in the last 20 years.

We compare the GrIS melt from our ANNs to melt calculated by the Modele Atmospherique Regional (MAR, Hanna et al., 2020), one of the most advanced RCM for studying GrIS surface processes, which has been extensively evaluated for studies of the GrIS (Fettweis et al., 2017). The aim is to test the quality of the melt estimate from ANNs. MAR dynamically downscaled a historical and SSP5‐8.5 (plus SSP1‐2.6 for CNRM‐CM‐6‐1) climate simulation from five global climate models (Figure 2). By comparing the melt predicted by each of the ANNs and the simulated melt from MAR, we find that the ANNs using $Z_{500}$, CC, and ${RAD}_{in}$ did not perform well (Figure S4). Likely, the reasons are that these variables are known to be uncertain in projections and dependent on the model physics. If an atmospheric variable is simulated very differently in the RCM versus the global climate model used for forcing, the relationship between this variable and melt in the ANN may not correspond to the relationship in the RCM. On the other hand, $T_{2m}$ and SNOW are likely more influenced by large‐scale dynamics, which will not differ much in the RCM compared to the global model used as lateral forcing (Ettema et al., 2010). Therefore, further in this comparison, we use the mean of the melt produced by the ANNs trained with $T_{2m}$ and SNOW.

The ANNs' integrated melt correlates very well to the melt simulated by MAR for all the different climate models (Figures 2b–2e). The mean of all the models (Figure 2f) shows a correlation as strong as the correlation using CESM2. Except for CNRM‐ESM2‐1, the interannual melt variability simulated by MAR is also well captured by the ANNs. The RMSE for individual SSP5‐8.5 simulations is in the range of 8.4–16.1% (60.0% of the SSP1‐2.6 simulation). The primary cause of this error is the ANNs producing lower interannual melt variability magnitude due to the ANNs being a mean of the ANN for $T_{2m}$ and SNOW. The RMSE for the mean of all models (both for MAR and the ANNs) is lower than for individual simulations.

For CESM2, the ANNs generate an end‐of‐the‐century mean melt that is, 15.6% lower than what is simulated by MAR. Keeping in mind that the ANNs captured this metric well for CESM2, this bias's leading cause is the difference in melt simulated by MAR and CESM2 for the same (global) climate simulation. The models with lower climate sensitivity than CESM2 (CNRM‐CM6‐1, CNRM‐ESM2‐1, and MRI‐ESM2‐0), the ANNs have a positive biased end‐of‐the‐century melt projection compared to MAR. The model with higher climate sensitivity than CESM2 (UKESM1‐0‐LL) has a negative bias compared to MAR, although smaller (−1.7%) than the CESM2 bias. The percentage bias for the SSP1‐2.6 is much larger than for the SSP5‐8.5, with the ANNs projecting a 71.9% higher melt increase than MAR for the SSP1‐2.6 simulation. When comparing the mean of all the SSP5‐8.5 simulations with MAR and the ANNs, the bias is small (1.6%; Figure 2f), and the MAR simulated melt is within the ANNs' model spread.

### 3.3 Surface Melt Projections

**Figure 3:**
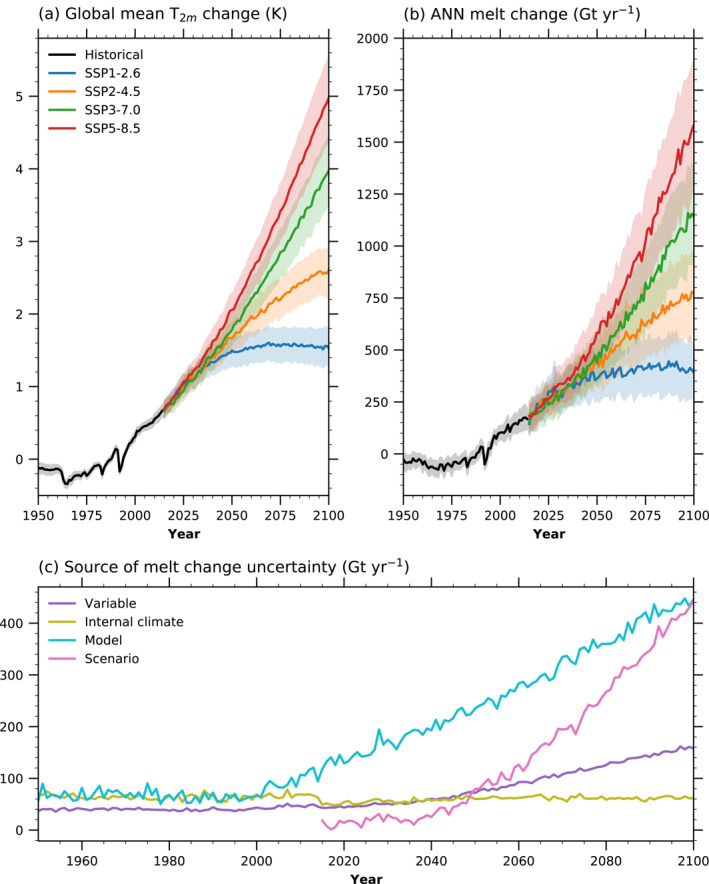
Climate Model Intercomparison Project Phase 6 (CMIP6) mean global mean temperature and Greenland ice sheet (GrIS) surface melt change in the historical period and the four Shared Socioeconomic Pathways (SSPs) for (a) $T_{2m}$ change (*k*) and (b) GrIS surface melt change (Gt ${yr}^{-1}$) with respect to the 1979–1998 mean. (c) Source of melt change uncertainty in the projections. For panels (a and b), black lines show the historical period, blue lines show SSP1‐2.6, orange lines show SSP2‐4.5, green lines show SSP3‐7.0, and red lines show SSP5‐8.5. The shading represents the model spread at ±0.5 standard deviation (instead of 1 standard deviation to avoid heavy overlapping). In panel (c), the purple line corresponds to the variable spread, olive line to internal climate spread, cyan line to model spread, and pink line to scenario spread. The spread is given by one standard deviation. Further details on uncertainty quantification are given in Text S1.

The global mean $T_{2m}$ evolution for the CMIP6 model ensemble (Table S2) using 1979–1998 as a baseline is shown in Figure 3a. The ensemble mean shows increasing temperatures since the 1960s (Figure 3a). All the scenarios show significant (p<0.01) warming in the last 20 years of the simulation (Table 1), except for SSP1‐2.6.

**Table 1:** Surface Melt Projections From Community Earth System Model 2.1 (CESM2) (Explicitly Simulated) and the Artificial Neural Networks (ANNs) Using the Mean of $T_{2m}$ and SNOW

Scenario	Global mean $T_{2m}$	CESM2	ANNs evaluated on CMIP6
Historical (mean)	16.0 ± 0.1	447 ± 90	521 ± 63
SSP1‐2.6 (change)	1.6 ± 0.5	413 ± 95	414 ± 276
SSP2‐4.5 (change)	2.5 ± 0.6	619 ± 140	724 ± 371
SSP3‐7.0 (change)	3.5 ± 0.8	1,040 ± 170	1,031 ± 436
SSP5‐8.5 (change)	4.4 ± 1.0	1,834 ± 152	1,378 ± 555

*Note*. “Historical” represents the 1979–1998 mean. For SSP's, 2081–2100 anomalies with respect to Historical are given. The ± denotes one standard deviation model spread for the last 20 years. The column ANNs gives the results for the full range of CMIP6 models. With the exception of the global mean $T_{2m}$ (K), all numbers are of GrIS surface melt (Gt ${yr}^{-1}$).

Using the $T_{2m}$ and SNOW ANNs, the global warming simulation can be translated into GrIS surface melt (Figure 3b and Table 1). For the historical period, surface melt starts to increase around 1975. The ANNs project the melt to increase in the range of 79% (SSP1‐2.6) to 264% (SSP5‐8.5) (Table 1) with respect to the 1979–1998 mean. Surface melt diverges between the years 2030 and 2050 for the different scenarios. By the end of the century, all SSP scenarios except for SSP1‐2.6 show a significant positive trend, meaning they will likely continue to increase beyond the 21st century. SSP1‐2.6 shows a nonsignificant (p = 0.02) decrease. Generally, the ANNs project a higher surface melt increase for models with a higher increase in global mean temperature (Figure S5).

The projected surface melt increase by the ANNs agrees well with the explicitly simulated melt increase by CESM2 (Table 1) for all scenarios except for SSP5‐8.5. This difference is likely due to the large range of climate sensitivities of the CMIP6 models (Zelinka et al., 2020), augmented by the different regional simulations for a given global mean temperature increase. Also, the ANNs project higher uncertainties for each scenario than CESM2.

The primary source of projection uncertainty is the climate model spread (Figure 3c), defined as the standard deviation of the climate model mean melt calculated by the ANNs (Text S1). The climate models' different climate sensitivity (Figure S5) is the primary cause of the large spread. The uncertainty related to scenario spread (defined as the standard deviation of the scenario's mean melt) is smaller than the model spread throughout the 21st century but becomes similar in the last years. The uncertainties associated with internal climate variability and variable selection ($T_{2m}$ and SNOW) are smaller than the sources mentioned above. While the spread due to internal climate variability remains relatively constant, the spread due to variable selection increases from 2040.

## 4 Discussion and Conclusions

In this study, we have trained ANNs with data from a global climate model, which includes an explicit simulation of ice sheets SMB (CESM2) to simulate 21st century GrIS surface melt from the atmospheric output of the full CMIP6 suite of climate models. The ANNs show good performance in predicting melt compared to the explicit modeling in an independent CESM2 simulation and simulations with the RCM MAR for a subset of CMIP6 models. By using the full suite of CMIP6 models, we can reassess the uncertainty of future melt estimates. Our results provide a larger uncertainty (+87 Gt ${yr}^{-1}$) for the melt corresponding to the SSP5‐8.5 scenario than a recent estimate from RCMs (Hanna et al., 2020), likely due to the inclusion of additional climate models and simulations per scenario. We find that the model spread is the primary source of uncertainty in the projections. By the end of the century, uncertainty due to scenario spread becomes an equal contributor to projection uncertainty.

The ANNs project GrIS surface melt increase by 414 ± 276, 724 ± 371, 1,031 ± 436, and 1,378 ± 555 Gt ${yr}^{-1}$ for SSP1‐2.6, SSP2‐4.5, SSP3‐7.0, and SSP5‐8.5, respectively. These projected increases here are higher than previous estimates, which give melt increases of 648 Gt ${yr}^{-1}$ (van Angelen et al., 2013) for Representative Concentration Pathway (RCP) 4.5 and 634 Gt ${yr}^{-1}$ for RCP8.5 (Vizcaino et al., 2014). The higher melt increases projected here are likely connected with the higher climate sensitivity of the CMIP6 models when compared to the CMIP5 models (Meehl et al., 2020; Zelinka et al., 2020).

We opted for a simple ANN structure in this study, as it allows us to explore the feature maps and activations easily. More advanced ANN structures, like multichannel deep convolutional neural networks (Barros et al., 2014), which can draw connections between variables, could be applied in a follow‐up for this study for comparison of projections.

## Supporting information

Supporting Information S1

## Data Availability

Projected melt from the ANNs can be obtained from https://doi.org/10.5281/zenodo.4424895, and the code used for this manuscript, as well as the models, are available from https://doi.org/10.5281/zenodo.5153908.
