# Supplementary material for: First Application of Artificial Neural Networks to Estimate 21st Century Greenland Ice Sheet Surface Melt
Source: Geophys Res Lett. 2021 Aug 19;48(16):e2021GL092449. doi: 10.1029/2021GL092449 (PMC9285918; doi:10.1029/2021GL092449)
Supplement: Supplementary file 1 — Supporting Information S1 [file GRL-48-0-s001.pdf]

Supporting Information for "First application of artificial neural networks to estimate 21st century Greenland ice sheet surface melt"

Raymond Sellevold[1]and Miren Vizcaino[1]

[1]Geoscience and remote sening, Delft University of Technology, Delft, the Netherlands

Contents of this file

1. Text S1
2. Figure S1
3. Figure S2
4. Figure S3
5. Figure S4
6. Figure S5
7. Table S1
8. Table S2

---

1. Uncertainty quantification

The uncertainties presented in Fig. 3c are calculated as follows:

- **Variable spread**: For each climate simulation, we calculate the standard deviation of the melt predicted by the T_{2m} ANN and the SNOW ANN. Then, we show the mean standard deviation over all climate simulations.
- **Internal climate spread**: We calculate the mean melt from the ANNs (T_{2m} and SNOW) for each simulation. Then, for each year, and by treating each scenario and model independently, we calculate the standard deviation of the melt rates of the simulations.
- **Model spread**: For each scenario, we take the standard deviation of the mean melt rates from the climate models. We show the mean standard deviation per year.
- **Scenario spread**: The standard deviation of the mean melt from each scenario is shown per year.

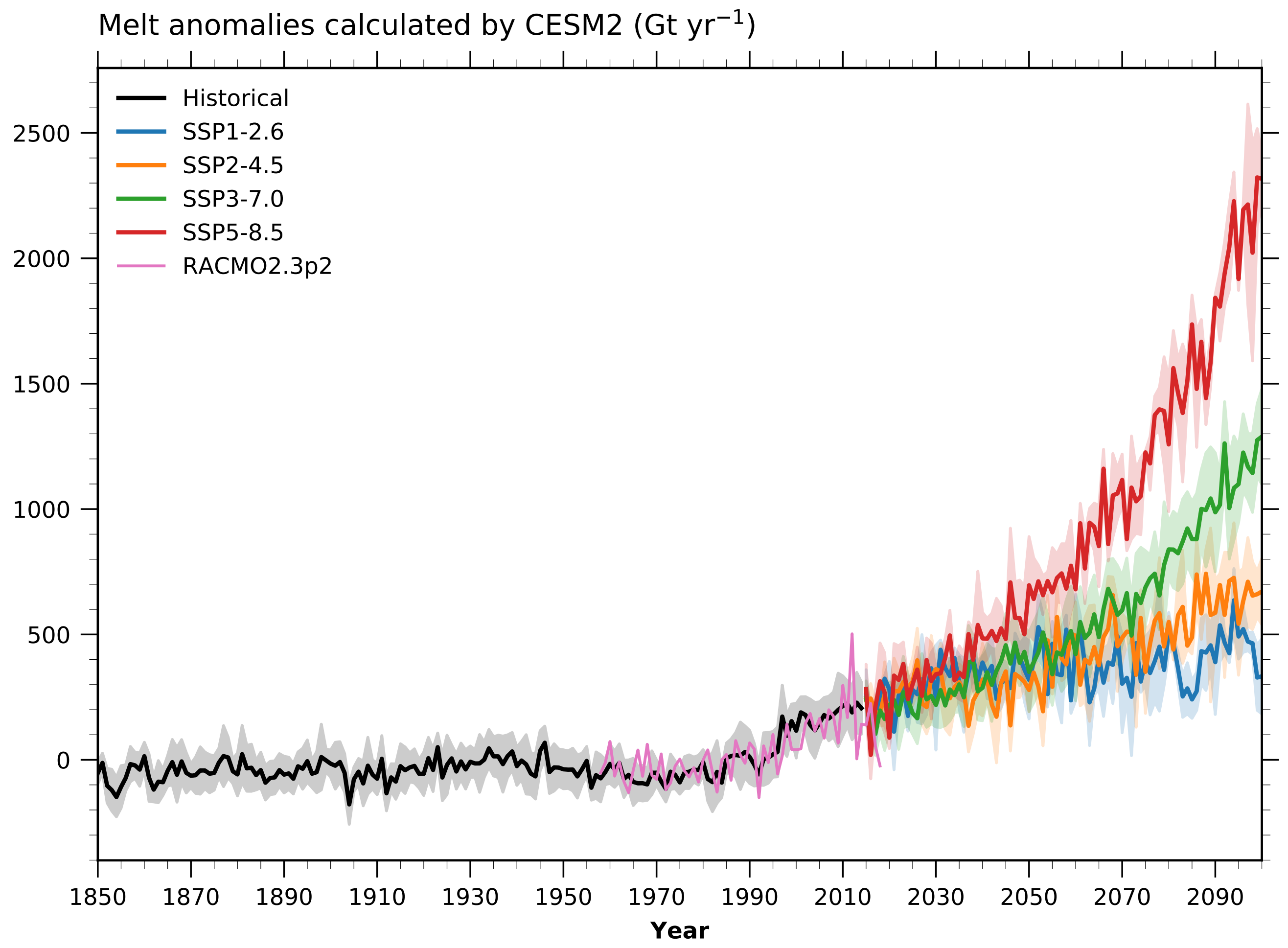

Figure S1. CESM2 ensemble mean melt anomalies (Gt yr^{-1}) in the years 1850–2100 with respect to 1979–1998. The lines shown are for the historical period (black; 10 ensembles), SSP1-2.6 (blue; 3 ensembles), SSP2-4.5 (orange; 3 ensembles), SSP3-7.0 (green; 10 ensembles), and SSP5-8.5 (red; 3 ensembles). The pink line represents the RCM-simulated melt (1958–2018) (Noël et al., 2020). Shading represents the ensemble spread at $\pm$ 1 standard deviation.

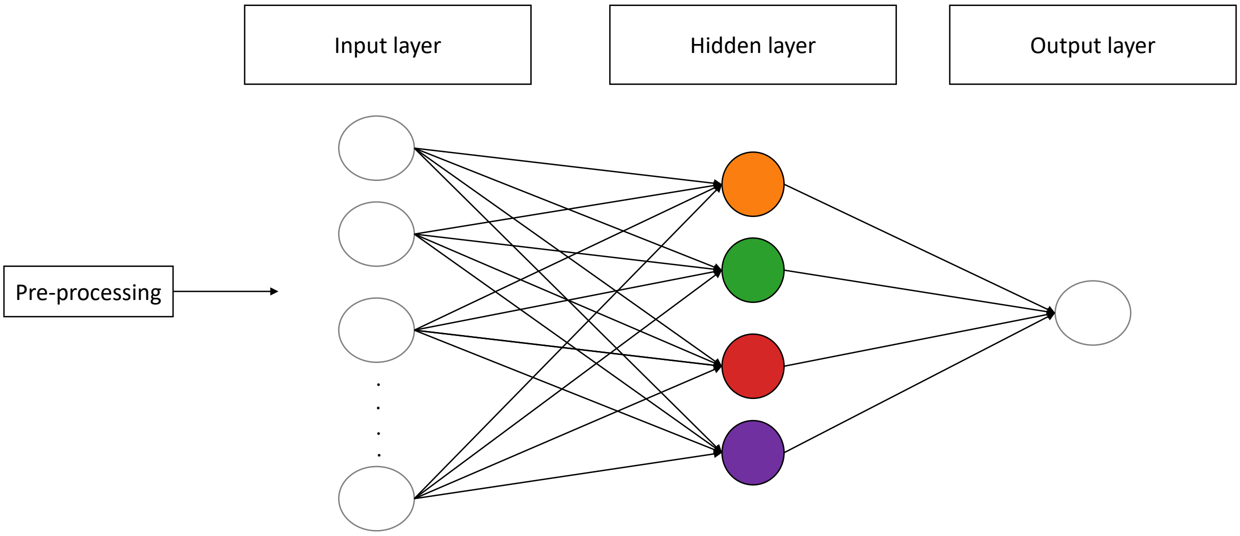

Figure S2. Schematic overview of the architecture for each of the neural networks. The colors of the hidden layer units correspond to the colors of the feature maps in Fig. 1 and Fig. S3.

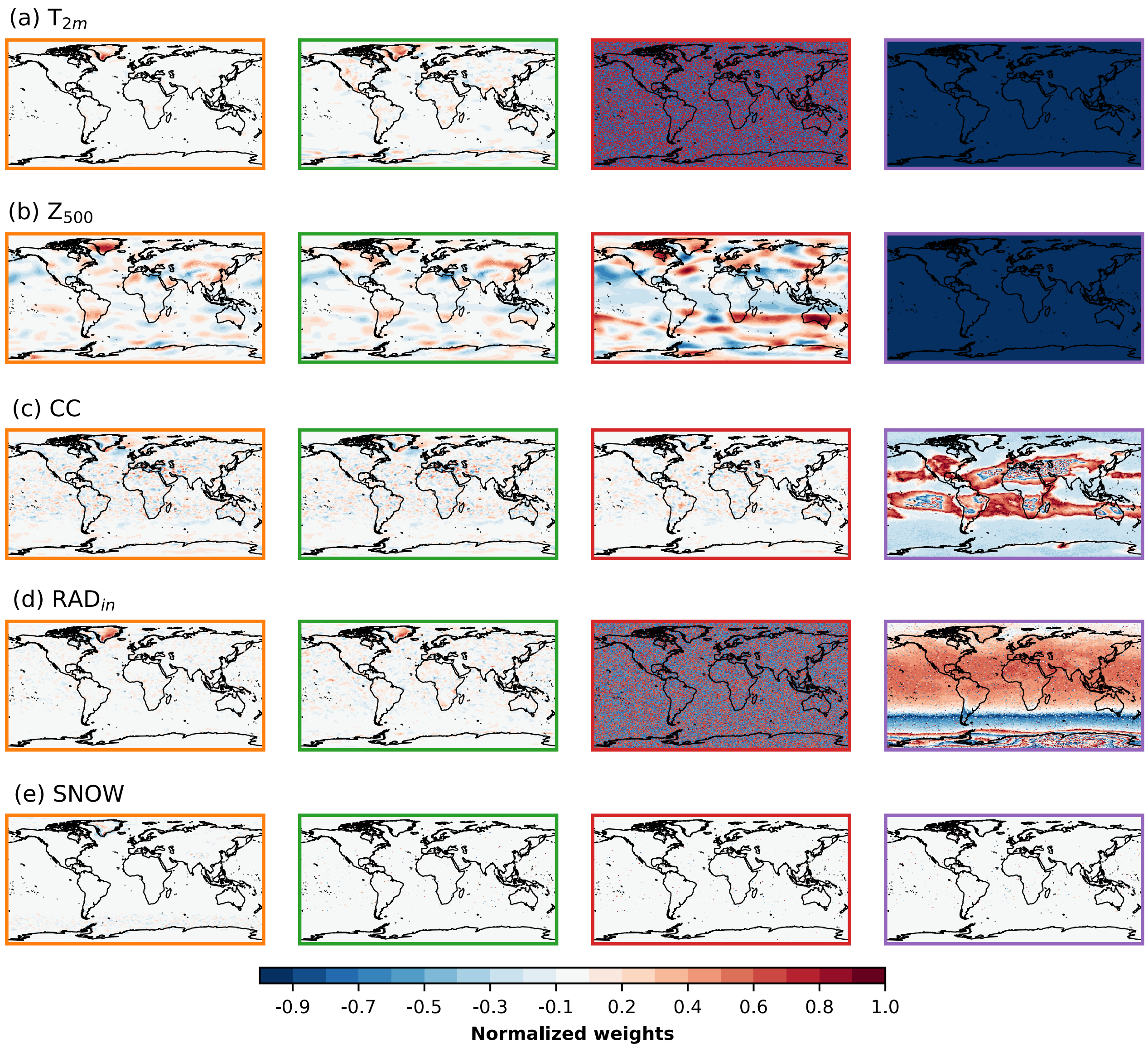

Figure S3. Global feature maps for the variables (a) near-surface temperature, (b) 500 hpa geopotential heights, (c) cloud cover, (d) incoming radiation, and (e) snowfall. The colored frame around the maps show which hidden unit each map is from (Fig. S2). Note: the green map for SNOW, the red maps for T_{2m}, RAD_{in}, and SNOW, as well as the purple maps are not activated by the neural networks.

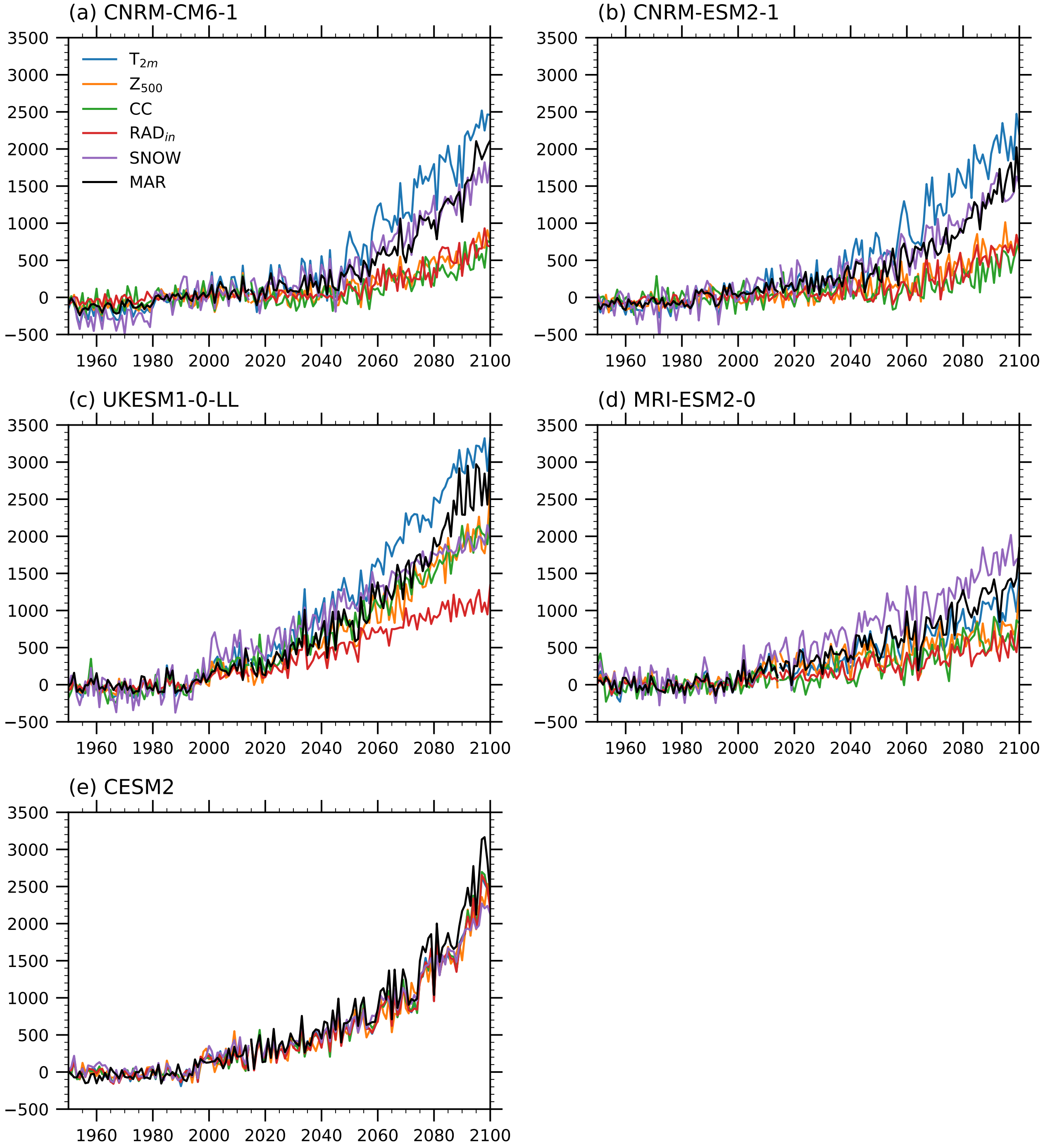

Figure S4. Surface melt predicted by the ANNs and simulated by MAR (Gt yr^{-1}) for a) CNRM-CM6-1, b) CNRM-ESM2-1, c) UKESM1-0-LL, d) MRI-ESM2-0, and e) CESM2, all for the SSP5-8.5. The black line shows the MAR simulated melt, the other lines show the ANN prediction using the variables: T_{2m} (blue), Z_{500} (orange), CC (green), RAD_{in} (red), and SNOW (purple).

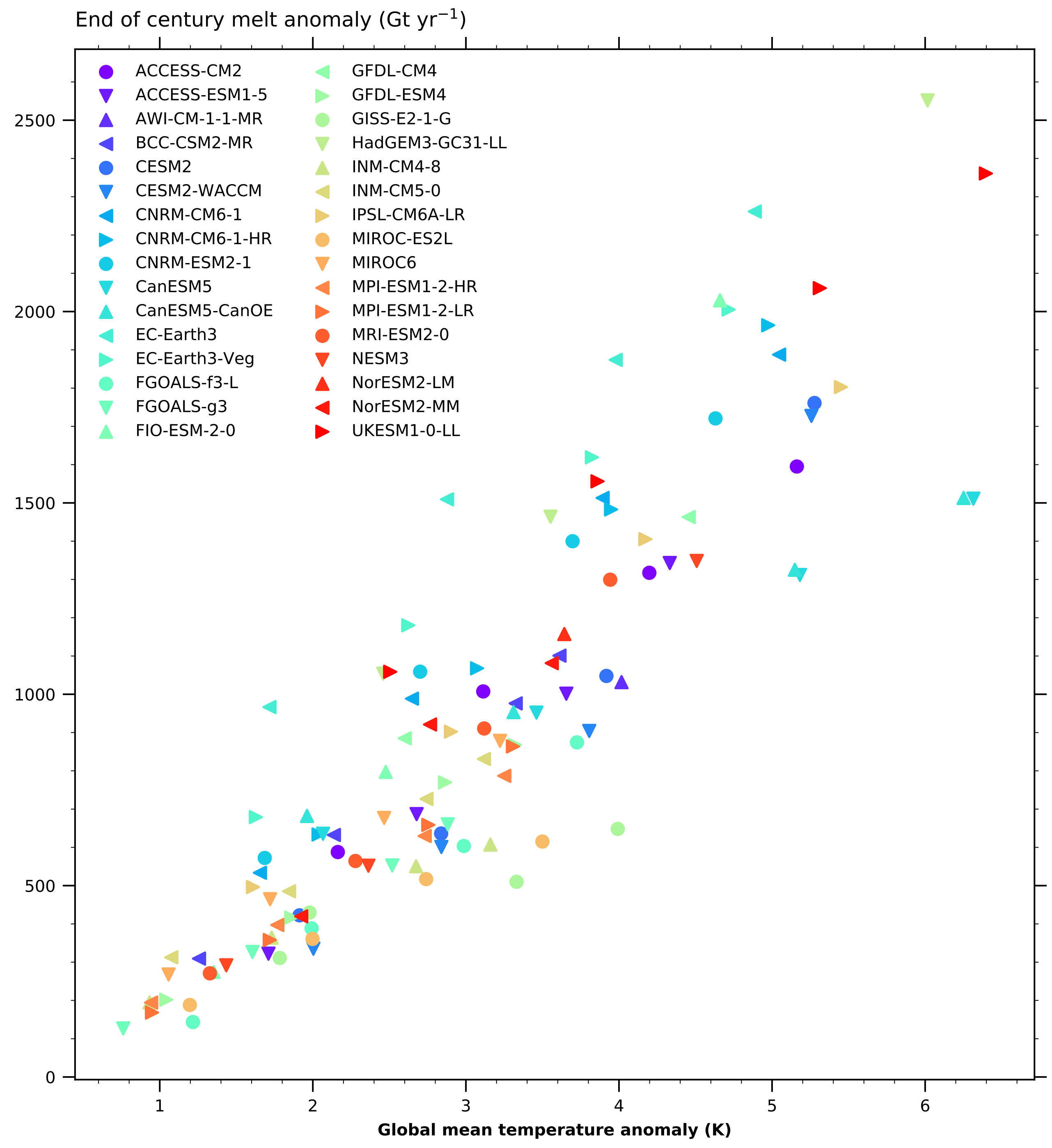

Figure S5. Ensemble mean 2081–2100 surface melt anomalies (Gt yr^{-1}) and global mean temperature anomalies (K), compared to a 1979–1998 climatology, for all the SSPs.

Table S1. Hyperparameters for the training of the artificial neural network. The scaling is the scaling applied to the input data, where X correponds to the variable values. λ is the regularization parameter applied to the hidden layer, and learning rate is the learning rate of the optimizer.

Variable	Epochs	Scaling	λ	Learning rate
T_{2m} [K]	19757	(X-180.0) / 160.0	10^{-2}	10^{-4}
Z_{500} [m]	5837	(X-4250.0) / 2000.0	10^{-1}	10^{-4}
CC [-]	630	X (no scaling)	10^{-2}	10^{-3}
RAD_{in} [W m^{-2}]	5000	X / 1000.0	10^{-1}	10^{-4}
SNOW [mm yr^{-1}]	1048	X / 7000.0	1	10^{-3}

Table S2: Number of ensembles per CMIP6 model used for the historical reconstruction and SSP projections of melt by the ANNs.

Model<br>*Insitution*	Historical	SSPs	Citation
ACCESS-CM2<br>*SCIRO-ARCCSS*	2	4	Dix et al. (2019a, 2019b, 2019c, 2019d, 2019e)
ACCESS-ESM1-5<br>*SCIRO*	3	12	Ziehn et al. (2019a, 2019b, 2019c, 2019d, 2019e)
AWI-CM-1-1-MR<br>*AWI*	1	8	Semmler et al. (2018a, 2018b, 2018c, 2019a, 2019b)
BCC-CSM2-MR<br>*BCC*	3	4	Wu et al. (2018); Xin et al. (2019a, 2019b, 2019c, 2019d)
BCC-ESM1<br>*BCC*	3		Zhang et al. (2018)
CAMS-CSM1-0<br>*CAMS*	2		Rong (2019)
CAS-ESM2-0<br>*CAS*	4		Chai (2020)
CESM2<br>*NCAR*	10	19	Danabasoglu (2019b, 2019c, 2019d, 2019e, 2019f)
CESM2-FV2<br>*NCAR*	3		Danabasoglu (2019a)
CESM2-WACCM<br>*NCAR*	3	12	Danabasoglu (2019h, 2019i, 2019j, 2019k, 2019l)
CESM2-WACCM-FV2<br>*NCAR*	3		Danabasoglu (2019g)
CNRM-CM6-1	30	24	Voldoire (2018, 2019d, 2019e, 2019f, 2019g)

CNRM-CERFACS			
CNRM-CM6-1-HR	1	4	Voldoire (2019a, 2020a, 2019b, 2020b, 2019c)
CNRM-CERFACS			
CNRM-ESM2-1	9	20	Seferian (2018); Voldoire (2019h, 2019i, 2019j, 2019k)
CNRM-CERFACS			
CanESM5	50	200	Swart et al. (2019f, 2019g, 2019h, 2019i, 2019j)
CCCma			
CanESM5-CanOE	3	12	Swart et al. (2019a, 2019b, 2019c, 2019d, 2019e)
CCCma			
E3SM-1-0	3		Bader, Leung, Taylor, and McCoy (2019a)
E3SM-Project			
E3SM-1-1	1		Bader, Leung, Taylor, and McCoy (2019b)
E3SM-Project			
E3SM-1-1-ECA	1		Bader, Leung, Taylor, and McCoy (2020)
E3SM-Project			
EC-Earth3	4	6	(EC-Earth) (2019a, 2019b, 2019c, 2019d, 2019e)
EC-Earth Consortium			
EC-Earth3-Veg	4	13	(EC-Earth) (2019f, 2019g, 2019h, 2019i, 2019j)
EC-Earth Consortium			
FGOALS-f3-L	3	4	YU (2019a, 2019b, 2019c, 2019d, 2019e)
CAS			
FGOALS-g3	5	4	Li (2019a, 2019b, 2019c, 2019d, 2019e)
CAS			
FIO-ESM-2-0	3	9	Song et al. (2019a, 2019b, 2019c, 2019d)
FIO-QLNM			
GFDL-CM4	1	2	Guo et al. (2018a, 2018b, 2018c)
NOAA-GFDL			

GFDL-ESM4	2	6	Krasting et al. (2018); John et al. (2018a, 2018b, 2018c, 2018d)
NOAA-GFDL			
GISS-E2-1-G	39	13	NASA/GISS (2018, 2020a, 2020b, 2020c, 2020d)
NASA-GISS			
GISS-E2-1-G-CC	1		NASA/GISS (2019a)
NASA-GISS			
GISS-E2-1-H	23		NASA/GISS (2019b)
NASA-GISS			
HadGEM3-GC31-LL	4	5	Ridley, Menary, Kuhlbrodt, Andrews, and Andrews (2019a); Good (2020a, 2019, 2020b)
MOHC			
HadGEM3-GC31-MM	2		Ridley, Menary, Kuhlbrodt, Andrews, and Andrews (2019b)
MOHC			
INM-CM4-8	1	4	Volodin et al. (2019a, 2019b, 2019c, 2019d, 2019e)
INM			
INM-CM5-0	10	8	Volodin et al. (2019f, 2019g, 2019h, 2019i, 2019j)
INM			
IPSL-CM6A-LR	32	34	Boucher, Denvil, Caubel, and Foujols (2018, 2019a, 2019b, 2019c, 2019d)
IPSL			
MIROC-ES2L	10	6	Hajima et al. (2019); Tachiiri et al. (2019a, 2019b, 2019c, 2019d)
MIROC			
MIROC6	10	12	Tatebe and Watanabe (2018); Shiogama, Abe, and Tatebe (2019a, 2019b, 2019c, 2019d)
MIROC			
MPI-ESM-1-2-HAM	2		Neubauer et al. (2019)

HAMMOZ-Consortium			
MPI-ESM1-2-HR	10	16	Jungclaus et al. (2019); Schupfner et al. (2019a, 2019b, 2019c, 2019d)
MPI-M			
MPI-ESM1-2-LR	10	40	Wieners et al. (2019a, 2019b, 2019c, 2019d, 2019e)
MPI-M			
MRI-ESM2-0	6	9	Yukimoto et al. (2019a, 2019b, 2019c, 2019d, 2019e)
MRI			
NESM3	5	6	Cao and Wang (2019); Cao (2019a, 2019b, 2019c)
NUIST			
NorESM2-LM	3	6	Seland et al. (2019a, 2019b, 2019c, 2019d, 2019e)
NCC			
NorESM2-MM	1	4	Bentsen et al. (2019a, 2019b, 2019c, 2019d, 2019e)
NCC			
SAM0-UNICON	1		Park and Shin (2019)
SNU			
TaiESM1	1		Lee and Liang (2020)
AS-RCEC			
UKESM1-0-LL	17	20	Tang et al. (2019); Good et al. (2019a, 2019b, 2019c, 2019d)
MOHC			
**Total**	**345**	**546**	

References

Bader, D. C., Leung, R., Taylor, M., & McCoy, R. B. (2019a). *E3SM-Project E3SM1.0 model output prepared for CMIP6 CMIP historical. Version 20200512.* Earth System Grid Federation. doi: 10.22033/ESGF/CMIP6.4497

Bader, D. C., Leung, R., Taylor, M., & McCoy, R. B. (2019b). *E3SM-Project E3SM1.1 model output prepared for CMIP6 CMIP historical. Version 20200512.* Earth System Grid Federation. doi: 10.22033/ESGF/CMIP6.11485

Bader, D. C., Leung, R., Taylor, M., & McCoy, R. B. (2020). *E3SM-Project E3SM1.1ECA model output prepared for CMIP6 CMIP historical. Version 20200512.* Earth System Grid Federation. doi: 10.22033/ESGF/CMIP6.11486

Bentsen, M., Olivié, D. J. L., Seland, y., Toniazzo, T., Gjermundsen, A., Graff, L. S., ... Schulz, M. (2019a). *NCC NorESM2-MM model output prepared for CMIP6 CMIP historical. Version 20200512.* Earth System Grid Federation. doi: 10.22033/ESGF/CMIP6.8040

Bentsen, M., Olivié, D. J. L., Seland, y., Toniazzo, T., Gjermundsen, A., Graff, L. S., ... Schulz, M. (2019b). *NCC NorESM2-MM model output prepared for CMIP6 ScenarioMIP ssp126. Version 20200512.* Earth System Grid Federation. doi: 10.22033/ESGF/CMIP6.8250

Bentsen, M., Olivié, D. J. L., Seland, y., Toniazzo, T., Gjermundsen, A., Graff, L. S., ... Schulz, M. (2019c). *NCC NorESM2-MM model output prepared for CMIP6 ScenarioMIP ssp245. Version 20200512.* Earth System Grid Federation. doi: 10.22033/ESGF/CMIP6.8255

Bentsen, M., Olivié, D. J. L., Seland, y., Toniazzo, T., Gjermundsen, A., Graff, L. S., ... Schulz, M. (2019d). *NCC NorESM2-MM model output prepared for CMIP6 ScenarioMIP ssp370. Version 20200512.* Earth System Grid Federation. doi: 10.22033/ESGF/CMIP6.8270

Bentsen, M., Olivié, D. J. L., Seland, y., Toniazzo, T., Gjermundsen, A., Graff, L. S., ... Schulz, M. (2019e). *NCC NorESM2-MM model output prepared for CMIP6 ScenarioMIP ssp585. Version 20200512.* Earth System Grid Federation. doi: 10.22033/ESGF/CMIP6.8321

Boucher, O., Denvil, S., Caubel, A., & Foujols, M. A. (2018). *IPSL IPSL-CM6A-LR model output prepared for CMIP6 CMIP historical. Version 20200512.* Earth System Grid Federation. doi: 10.22033/ESGF/CMIP6.5195

Boucher, O., Denvil, S., Caubel, A., & Foujols, M. A. (2019a). *IPSL IPSL-CM6A-LR model output prepared for CMIP6 ScenarioMIP ssp126. Version 20200512.* Earth System Grid Federation. doi: 10.22033/ESGF/CMIP6.5262

Boucher, O., Denvil, S., Caubel, A., & Foujols, M. A. (2019b). *IPSL IPSL-CM6A-LR model output prepared for CMIP6 ScenarioMIP ssp245. Version 20200512.* Earth System Grid Federation. doi: 10.22033/ESGF/CMIP6.5264

Boucher, O., Denvil, S., Caubel, A., & Foujols, M. A. (2019c). *IPSL IPSL-CM6A-LR model output prepared for CMIP6 ScenarioMIP ssp370. Version 20200512.* Earth System Grid Federation. doi: 10.22033/ESGF/CMIP6.5265

Boucher, O., Denvil, S., Caubel, A., & Foujols, M. A. (2019d). *IPSL IPSL-CM6A-LR model output prepared for CMIP6 ScenarioMIP ssp585. Version 20200512.* Earth System Grid Federation. doi: 10.22033/ESGF/CMIP6.5271

Cao, J. (2019a). *NUIST NESMv3 model output prepared for CMIP6 ScenarioMIP ssp126. Version 20200512.* Earth System Grid Federation. doi: 10.22033/ESGF/CMIP6.8780

Cao, J. (2019b). *NUIST NESMv3 model output prepared for CMIP6 ScenarioMIP ssp245. Version 20200512.* Earth System Grid Federation. doi: 10.22033/ESGF/CMIP6.8781

Cao, J. (2019c). *NUIST NESMv3 model output prepared for CMIP6 ScenarioMIP ssp585. Version 20200512.* Earth System Grid Federation. doi: 10.22033/ESGF/CMIP6.8790

Cao, J., & Wang, B. (2019). *NUIST NESMv3 model output prepared for CMIP6 CMIP historical. Version 20200512.* Earth System Grid Federation. doi: 10.22033/ESGF/CMIP6.8769

Chai, Z. (2020). *CAS CAS-ESM1.0 model output prepared for CMIP6 CMIP historical. Version 20200512.* Earth System Grid Federation. doi: 10.22033/ESGF/CMIP6.3353

Danabasoglu, G. (2019a). *NCAR CESM2-FV2 model output prepared for CMIP6 CMIP historical. Version 20200512.* Earth System Grid Federation. doi: 10.22033/ESGF/CMIP6.11297

Danabasoglu, G. (2019b). *NCAR CESM2 model output prepared for CMIP6 CMIP historical. Version 20200512.* Earth System Grid Federation. doi: 10.22033/ESGF/CMIP6.7627

Danabasoglu, G. (2019c). *NCAR CESM2 model output prepared for CMIP6 ScenarioMIP ssp126. Version 20200512.* Earth System Grid Federation. doi: 10.22033/ESGF/CMIP6.7746

Danabasoglu, G. (2019d). *NCAR CESM2 model output prepared for CMIP6 ScenarioMIP ssp245. Version 20200512.* Earth System Grid Federation. doi: 10.22033/ESGF/CMIP6.7748

Danabasoglu, G. (2019e). *NCAR CESM2 model output prepared for CMIP6 ScenarioMIP ssp370. Version 20200512.* Earth System Grid Federation. doi: 10.22033/ESGF/CMIP6.7753

Danabasoglu, G. (2019f). *NCAR CESM2 model output prepared for CMIP6 ScenarioMIP ssp585. Version 20200512.* Earth System Grid Federation. doi: 10.22033/ESGF/CMIP6.7768

Danabasoglu, G. (2019g). *NCAR CESM2-WACCM-FV2 model output prepared for CMIP6 CMIP historical. Version 20200512.* Earth System Grid Federation. doi: 10.22033/ESGF/CMIP6.11298

Danabasoglu, G. (2019h). *NCAR CESM2-WACCM model output prepared for CMIP6 CMIP historical. Version 20200512.* Earth System Grid Federation. doi: 10.22033/ESGF/CMIP6.10071

Danabasoglu, G. (2019i). *NCAR CESM2-WACCM model output prepared for CMIP6 ScenarioMIP ssp126. Version 20200512.* Earth System Grid Federation. doi: 10.22033/ESGF/CMIP6.10100

Danabasoglu, G. (2019j). *NCAR CESM2-WACCM model output prepared for CMIP6 ScenarioMIP ssp245. Version 20200512.* Earth System Grid Federation. doi: 10.22033/ESGF/CMIP6.10101

Danabasoglu, G. (2019k). *NCAR CESM2-WACCM model output prepared for CMIP6 ScenarioMIP ssp370. Version 20200512.* Earth System Grid Federation. doi: 10.22033/ESGF/CMIP6.10102

Danabasoglu, G. (2019l). *NCAR CESM2-WACCM model output prepared for CMIP6 ScenarioMIP ssp585. Version 20200512.* Earth System Grid Federation. doi: 10.22033/ESGF/CMIP6.10115

Dix, M., Bi, D., Dobrohotoff, P., Fiedler, R., Harman, I., Law, R., ... Yang, R. (2019a). *CSIRO-ARCCSS ACCESS-CM2 model output prepared for CMIP6 CMIP historical. Version 20200512.* Earth System Grid Federation. doi: 10.22033/ESGF/CMIP6.4271

Dix, M., Bi, D., Dobrohotoff, P., Fiedler, R., Harman, I., Law, R., ... Yang, R. (2019b).

CSIRO-ARCCSS ACCESS-CM2 model output prepared for CMIP6 ScenarioMIP ssp126. Version 20200512. Earth System Grid Federation. doi: 10.22033/ESGF/CMIP6.4319

Dix, M., Bi, D., Dobrohotoff, P., Fiedler, R., Harman, I., Law, R., ... Yang, R. (2019c). *CSIRO-ARCCSS ACCESS-CM2 model output prepared for CMIP6 ScenarioMIP ssp245. Version 20200512.* Earth System Grid Federation. doi: 10.22033/ESGF/CMIP6.4321

Dix, M., Bi, D., Dobrohotoff, P., Fiedler, R., Harman, I., Law, R., ... Yang, R. (2019d). *CSIRO-ARCCSS ACCESS-CM2 model output prepared for CMIP6 ScenarioMIP ssp370. Version 20200512.* Earth System Grid Federation. doi: 10.22033/ESGF/CMIP6.4323

Dix, M., Bi, D., Dobrohotoff, P., Fiedler, R., Harman, I., Law, R., ... Yang, R. (2019e). *CSIRO-ARCCSS ACCESS-CM2 model output prepared for CMIP6 ScenarioMIP ssp585. Version 20200512.* Earth System Grid Federation. doi: 10.22033/ESGF/CMIP6.4332

(EC-Earth), E.-E. C. (2019a). *EC-Earth-Consortium EC-Earth3 model output prepared for CMIP6 CMIP historical. Version 20200512.* Earth System Grid Federation. doi: 10.22033/ESGF/CMIP6.4700

(EC-Earth), E.-E. C. (2019b). *EC-Earth-Consortium EC-Earth3 model output prepared for CMIP6 ScenarioMIP ssp126. Version 20200512.* Earth System Grid Federation. doi: 10.22033/ESGF/CMIP6.4874

(EC-Earth), E.-E. C. (2019c). *EC-Earth-Consortium EC-Earth3 model output prepared for CMIP6 ScenarioMIP ssp245. Version 20200512.* Earth System Grid Federation. doi: 10.22033/ESGF/CMIP6.4880

(EC-Earth), E.-E. C. (2019d). *EC-Earth-Consortium EC-Earth3 model output prepared for CMIP6 ScenarioMIP ssp370. Version 20200512.* Earth System Grid Federation. doi: 10

.22033/ESGF/CMIP6.4884

(EC-Earth), E.-E. C. (2019e). *EC-Earth-Consortium EC-Earth3 model output prepared for CMIP6 ScenarioMIP ssp585. Version 20200512.* Earth System Grid Federation. doi: 10.22033/ESGF/CMIP6.4912

(EC-Earth), E.-E. C. (2019f). *EC-Earth-Consortium EC-Earth3-Veg model output prepared for CMIP6 CMIP historical. Version 20200512.* Earth System Grid Federation. doi: 10.22033/ESGF/CMIP6.4706

(EC-Earth), E.-E. C. (2019g). *EC-Earth-Consortium EC-Earth3-Veg model output prepared for CMIP6 ScenarioMIP ssp126. Version 20200512.* Earth System Grid Federation. doi: 10.22033/ESGF/CMIP6.4876

(EC-Earth), E.-E. C. (2019h). *EC-Earth-Consortium EC-Earth3-Veg model output prepared for CMIP6 ScenarioMIP ssp245. Version 20200512.* Earth System Grid Federation. doi: 10.22033/ESGF/CMIP6.4882

(EC-Earth), E.-E. C. (2019i). *EC-Earth-Consortium EC-Earth3-Veg model output prepared for CMIP6 ScenarioMIP ssp370. Version 20200512.* Earth System Grid Federation. doi: 10.22033/ESGF/CMIP6.4886

(EC-Earth), E.-E. C. (2019j). *EC-Earth-Consortium EC-Earth3-Veg model output prepared for CMIP6 ScenarioMIP ssp585. Version 20200512.* Earth System Grid Federation. doi: 10.22033/ESGF/CMIP6.4914

Good, P. (2019). *MOHC HadGEM3-GC31-LL model output prepared for CMIP6 ScenarioMIP ssp245. Version 20200512.* Earth System Grid Federation. doi: 10.22033/ESGF/CMIP6.10851

Good, P. (2020a). *MOHC HadGEM3-GC31-LL model output prepared for CMIP6 ScenarioMIP ssp126. Version 20200512.* Earth System Grid Federation. doi: 10.22033/ESGF/CMIP6.10849

Good, P. (2020b). *MOHC HadGEM3-GC31-LL model output prepared for CMIP6 ScenarioMIP ssp585. Version 20200512.* Earth System Grid Federation. doi: 10.22033/ESGF/CMIP6.10901

Good, P., Sellar, A., Tang, Y., Rumbold, S., Ellis, R., Kelley, D., & Kuhlbrodt, T. (2019a). *MOHC UKESM1.0-LL model output prepared for CMIP6 ScenarioMIP ssp126. Version 20200512.* Earth System Grid Federation. doi: 10.22033/ESGF/CMIP6.6333

Good, P., Sellar, A., Tang, Y., Rumbold, S., Ellis, R., Kelley, D., & Kuhlbrodt, T. (2019b). *MOHC UKESM1.0-LL model output prepared for CMIP6 ScenarioMIP ssp245. Version 20200512.* Earth System Grid Federation. doi: 10.22033/ESGF/CMIP6.6339

Good, P., Sellar, A., Tang, Y., Rumbold, S., Ellis, R., Kelley, D., & Kuhlbrodt, T. (2019c). *MOHC UKESM1.0-LL model output prepared for CMIP6 ScenarioMIP ssp370. Version 20200512.* Earth System Grid Federation. doi: 10.22033/ESGF/CMIP6.6347

Good, P., Sellar, A., Tang, Y., Rumbold, S., Ellis, R., Kelley, D., & Kuhlbrodt, T. (2019d). *MOHC UKESM1.0-LL model output prepared for CMIP6 ScenarioMIP ssp585. Version 20200512.* Earth System Grid Federation. doi: 10.22033/ESGF/CMIP6.6405

Guo, H., John, J. G., Blanton, C., McHugh, C., Nikonov, S., Radhakrishnan, A., . . . Zhang, R. (2018a). *NOAA-GFDL GFDL-CM4 model output historical. Version 20200512.* Earth System Grid Federation. doi: 10.22033/ESGF/CMIP6.8594

Guo, H., John, J. G., Blanton, C., McHugh, C., Nikonov, S., Radhakrishnan, A., . . . Zhang, R.

(2018b). *NOAA-GFDL GFDL-CM4 model output prepared for CMIP6 ScenarioMIP ssp245. Version 20200512.* Earth System Grid Federation. doi: 10.22033/ESGF/CMIP6.9263

Guo, H., John, J. G., Blanton, C., McHugh, C., Nikonov, S., Radhakrishnan, A., ... Zhang, R. (2018c). *NOAA-GFDL GFDL-CM4 model output prepared for CMIP6 ScenarioMIP ssp585. Version 20200512.* Earth System Grid Federation. doi: 10.22033/ESGF/CMIP6.9268

Hajima, T., Abe, M., Arakawa, O., Suzuki, T., Komuro, Y., Ogura, T., ... Tachiiri, K. (2019). *MIROC MIROC-ES2L model output prepared for CMIP6 CMIP historical. Version 20200512.* Earth System Grid Federation. doi: 10.22033/ESGF/CMIP6.5602

John, J. G., Blanton, C., McHugh, C., Radhakrishnan, A., Rand, K., Vahlenkamp, H., ... Zeng, Y. (2018a). *NOAA-GFDL GFDL-ESM4 model output prepared for CMIP6 ScenarioMIP ssp126. Version 20200512.* Earth System Grid Federation. doi: 10.22033/ESGF/CMIP6.8684

John, J. G., Blanton, C., McHugh, C., Radhakrishnan, A., Rand, K., Vahlenkamp, H., ... Zeng, Y. (2018b). *NOAA-GFDL GFDL-ESM4 model output prepared for CMIP6 ScenarioMIP ssp245. Version 20200512.* Earth System Grid Federation. doi: 10.22033/ESGF/CMIP6.8686

John, J. G., Blanton, C., McHugh, C., Radhakrishnan, A., Rand, K., Vahlenkamp, H., ... Zeng, Y. (2018c). *NOAA-GFDL GFDL-ESM4 model output prepared for CMIP6 ScenarioMIP ssp370. Version 20200512.* Earth System Grid Federation. doi: 10.22033/ESGF/CMIP6.8691

John, J. G., Blanton, C., McHugh, C., Radhakrishnan, A., Rand, K., Vahlenkamp, H., ... Zeng, Y. (2018d). *NOAA-GFDL GFDL-ESM4 model output prepared for CMIP6 ScenarioMIP*

ssp585. Version 20200512. Earth System Grid Federation. doi: 10.22033/ESGF/CMIP6.8706

Jungclaus, J., Bittner, M., Wieners, K.-H., Wachsmann, F., Schupfner, M., Legutke, S., ... Roeckner, E. (2019). *MPI-M MPI-ESM1.2-HR model output prepared for CMIP6 CMIP historical. Version 20200512.* Earth System Grid Federation. doi: 10.22033/ESGF/CMIP6.6594

Krasting, J. P., John, J. G., Blanton, C., McHugh, C., Nikonov, S., Radhakrishnan, A., ... Zhao, M. (2018). *NOAA-GFDL GFDL-ESM4 model output prepared for CMIP6 CMIP historical. Version 20200512.* Earth System Grid Federation. doi: 10.22033/ESGF/CMIP6.8597

Lee, W.-L., & Liang, H.-C. (2020). *AS-RCEC TaiESM1.0 model output prepared for CMIP6 CMIP historical. Version 20200512.* Earth System Grid Federation. doi: 10.22033/ESGF/CMIP6.9755

Li, L. (2019a). *CAS FGOALS-g3 model output prepared for CMIP6 CMIP historical. Version 20200512.* Earth System Grid Federation. doi: 10.22033/ESGF/CMIP6.3356

Li, L. (2019b). *CAS FGOALS-g3 model output prepared for CMIP6 ScenarioMIP ssp126. Version 20200512.* Earth System Grid Federation. doi: 10.22033/ESGF/CMIP6.3465

Li, L. (2019c). *CAS FGOALS-g3 model output prepared for CMIP6 ScenarioMIP ssp245. Version 20200512.* Earth System Grid Federation. doi: 10.22033/ESGF/CMIP6.3469

Li, L. (2019d). *CAS FGOALS-g3 model output prepared for CMIP6 ScenarioMIP ssp370. Version 20200512.* Earth System Grid Federation. doi: 10.22033/ESGF/CMIP6.3480

Li, L. (2019e). *CAS FGOALS-g3 model output prepared for CMIP6 ScenarioMIP ssp585. Version 20200512.* Earth System Grid Federation. doi: 10.22033/ESGF/CMIP6.3503

NASA/GISS. (2018). *NASA-GISS GISS-E2.1G model output prepared for CMIP6 CMIP historical. Version 20200512.* Earth System Grid Federation. doi: 10.22033/ESGF/CMIP6.7127

NASA/GISS. (2019a). *NASA-GISS GISS-E2-1-G-CC model output prepared for CMIP6 CMIP historical. Version 20200512.* Earth System Grid Federation. doi: 10.22033/ESGF/CMIP6.11762

NASA/GISS. (2019b). *NASA-GISS GISS-E2.1H model output prepared for CMIP6 CMIP historical. Version 20200512.* Earth System Grid Federation. doi: 10.22033/ESGF/CMIP6.7128

NASA/GISS. (2020a). *NASA-GISS GISS-E2.1G model output prepared for CMIP6 ScenarioMIP ssp126. Version 20200512.* Earth System Grid Federation. doi: 10.22033/ESGF/CMIP6.7410

NASA/GISS. (2020b). *NASA-GISS GISS-E2.1G model output prepared for CMIP6 ScenarioMIP ssp245. Version 20200512.* Earth System Grid Federation. doi: 10.22033/ESGF/CMIP6.7415

NASA/GISS. (2020c). *NASA-GISS GISS-E2.1G model output prepared for CMIP6 ScenarioMIP ssp370. Version 20200512.* Earth System Grid Federation. doi: 10.22033/ESGF/CMIP6.7426

NASA/GISS. (2020d). *NASA-GISS GISS-E2.1G model output prepared for CMIP6 ScenarioMIP ssp585. Version 20200512.* Earth System Grid Federation. doi: 10.22033/ESGF/CMIP6.7460

Neubauer, D., Ferrachat, S., Siegenthaler-Le Drian, C., Stoll, J., Folini, D. S., Tegen, I., … Lohmann, U. (2019). *HAMMOZ-Consortium MPI-ESM1.2-HAM model output prepared for*

CMIP6 CMIP historical. Version 20200512. Earth System Grid Federation. doi: 10.22033/ESGF/CMIP6.5016

Noël, B., van Kampenhout, L., van de Berg, W. J., Lenaerts, J. T. M., Wouters, B., & van den Broeke, M. R. (2020). Brief communication: CESM2 climate forcing (1950–2014) yields realistic Greenland ice sheet surface mass balance. *The Cryosphere*, *14*(4), 1425–1435. doi: 10.5194/tc-14-1425-2020

Park, S., & Shin, J. (2019). *SNU SAM0-UNICON model output prepared for CMIP6 CMIP historical. Version 20200512.* Earth System Grid Federation. doi: 10.22033/ESGF/CMIP6.7789

Ridley, J., Menary, M., Kuhlbrodt, T., Andrews, M., & Andrews, T. (2019a). *MOHC HadGEM3-GC31-LL model output prepared for CMIP6 CMIP historical. Version 20200512.* Earth System Grid Federation. doi: 10.22033/ESGF/CMIP6.6109

Ridley, J., Menary, M., Kuhlbrodt, T., Andrews, M., & Andrews, T. (2019b). *MOHC HadGEM3-GC31-MM model output prepared for CMIP6 CMIP historical. Version 20200512.* Earth System Grid Federation. doi: 10.22033/ESGF/CMIP6.6112

Rong, X. (2019). *CAMS CAMS-CSM1.0 model output prepared for CMIP6 CMIP historical. Version 20200512.* Earth System Grid Federation. doi: 10.22033/ESGF/CMIP6.9754

Schupfner, M., Wieners, K.-H., Wachsmann, F., Steger, C., Bittner, M., Jungclaus, J., … Roeckner, E. (2019a). *DKRZ MPI-ESM1.2-HR model output prepared for CMIP6 ScenarioMIP ssp126. Version 20200512.* Earth System Grid Federation. doi: 10.22033/ESGF/CMIP6.4397

Schupfner, M., Wieners, K.-H., Wachsmann, F., Steger, C., Bittner, M., Jungclaus, J., …

Roeckner, E. (2019b). *DKRZ MPI-ESM1.2-HR model output prepared for CMIP6 ScenarioMIP ssp245. Version 20200512.* Earth System Grid Federation. doi: 10.22033/ESGF/CMIP6.4398

Schupfner, M., Wieners, K.-H., Wachsmann, F., Steger, C., Bittner, M., Jungclaus, J., … Roeckner, E. (2019c). *DKRZ MPI-ESM1.2-HR model output prepared for CMIP6 ScenarioMIP ssp370. Version 20200512.* Earth System Grid Federation. doi: 10.22033/ESGF/CMIP6.4399

Schupfner, M., Wieners, K.-H., Wachsmann, F., Steger, C., Bittner, M., Jungclaus, J., … Roeckner, E. (2019d). *DKRZ MPI-ESM1.2-HR model output prepared for CMIP6 ScenarioMIP ssp585. Version 20200512.* Earth System Grid Federation. doi: 10.22033/ESGF/CMIP6.4403

Seferian, R. (2018). *CNRM-CERFACS CNRM-ESM2-1 model output prepared for CMIP6 CMIP historical. Version 20200512.* Earth System Grid Federation. doi: 10.22033/ESGF/CMIP6.4068

Seland, y., Bentsen, M., Oliviè, D. J. L., Toniazzo, T., Gjermundsen, A., Graff, L. S., … Schulz, M. (2019a). *NCC NorESM2-LM model output prepared for CMIP6 CMIP historical. Version 20200512.* Earth System Grid Federation. doi: 10.22033/ESGF/CMIP6.8036

Seland, y., Bentsen, M., Oliviè, D. J. L., Toniazzo, T., Gjermundsen, A., Graff, L. S., … Schulz, M. (2019b). *NCC NorESM2-LM model output prepared for CMIP6 ScenarioMIP ssp126. Version 20200512.* Earth System Grid Federation. doi: 10.22033/ESGF/CMIP6.8248

Seland, y., Bentsen, M., Oliviè, D. J. L., Toniazzo, T., Gjermundsen, A., Graff, L. S., … Schulz, M. (2019c). *NCC NorESM2-LM model output prepared for CMIP6 ScenarioMIP ssp245.*

Version 20200512. Earth System Grid Federation. doi: 10.22033/ESGF/CMIP6.8253

Seland, y., Bentsen, M., Oliviè, D. J. L., Toniazzo, T., Gjermundsen, A., Graff, L. S., … Schulz, M. (2019d). *NCC NorESM2-LM model output prepared for CMIP6 ScenarioMIP ssp370. Version 20200512.* Earth System Grid Federation. doi: 10.22033/ESGF/CMIP6.8268

Seland, y., Bentsen, M., Oliviè, D. J. L., Toniazzo, T., Gjermundsen, A., Graff, L. S., … Schulz, M. (2019e). *NCC NorESM2-LM model output prepared for CMIP6 ScenarioMIP ssp585. Version 20200512.* Earth System Grid Federation. doi: 10.22033/ESGF/CMIP6.8319

Semmler, T., Danilov, S., Rackow, T., Sidorenko, D., Barbi, D., Hegewald, J., … Jung, T. (2018a). *AWI AWI-CM1.1MR model output prepared for CMIP6 CMIP historical. Version 20200512.* Earth System Grid Federation. doi: 10.22033/ESGF/CMIP6.2686

Semmler, T., Danilov, S., Rackow, T., Sidorenko, D., Barbi, D., Hegewald, J., … Jung, T. (2018b). *AWI AWI-CM1.1MR model output prepared for CMIP6 ScenarioMIP ssp126. Version 20200512.* Earth System Grid Federation. doi: 10.22033/ESGF/CMIP6.2796

Semmler, T., Danilov, S., Rackow, T., Sidorenko, D., Barbi, D., Hegewald, J., … Jung, T. (2018c). *AWI AWI-CM1.1MR model output prepared for CMIP6 ScenarioMIP ssp245. Version 20200512.* Earth System Grid Federation. doi: 10.22033/ESGF/CMIP6.2800

Semmler, T., Danilov, S., Rackow, T., Sidorenko, D., Barbi, D., Hegewald, J., … Jung, T. (2019a). *AWI AWI-CM1.1MR model output prepared for CMIP6 ScenarioMIP ssp370. Version 20200512.* Earth System Grid Federation. doi: 10.22033/ESGF/CMIP6.2803

Semmler, T., Danilov, S., Rackow, T., Sidorenko, D., Barbi, D., Hegewald, J., … Jung, T. (2019b). *AWI AWI-CM1.1MR model output prepared for CMIP6 ScenarioMIP ssp585. Version 20200512.* Earth System Grid Federation. doi: 10.22033/ESGF/CMIP6.2817

Shiogama, H., Abe, M., & Tatebe, H. (2019a). *MIROC MIROC6 model output prepared for CMIP6 ScenarioMIP ssp126. Version 20200512.* Earth System Grid Federation. doi: 10.22033/ESGF/CMIP6.5743

Shiogama, H., Abe, M., & Tatebe, H. (2019b). *MIROC MIROC6 model output prepared for CMIP6 ScenarioMIP ssp245. Version 20200512.* Earth System Grid Federation. doi: 10.22033/ESGF/CMIP6.5746

Shiogama, H., Abe, M., & Tatebe, H. (2019c). *MIROC MIROC6 model output prepared for CMIP6 ScenarioMIP ssp370. Version 20200512.* Earth System Grid Federation. doi: 10.22033/ESGF/CMIP6.5752

Shiogama, H., Abe, M., & Tatebe, H. (2019d). *MIROC MIROC6 model output prepared for CMIP6 ScenarioMIP ssp585. Version 20200512.* Earth System Grid Federation. doi: 10.22033/ESGF/CMIP6.5771

Song, Z., Qiao, F., Bao, Y., Shu, Q., Song, Y., & Yang, X. (2019a). *FIO-QLNM FIO-ESM2.0 model output prepared for CMIP6 CMIP historical. Version 20200512.* Earth System Grid Federation. doi: 10.22033/ESGF/CMIP6.9199

Song, Z., Qiao, F., Bao, Y., Shu, Q., Song, Y., & Yang, X. (2019b). *FIO-QLNM FIO-ESM2.0 model output prepared for CMIP6 ScenarioMIP ssp126. Version 20200512.* Earth System Grid Federation. doi: 10.22033/ESGF/CMIP6.9208

Song, Z., Qiao, F., Bao, Y., Shu, Q., Song, Y., & Yang, X. (2019c). *FIO-QLNM FIO-ESM2.0 model output prepared for CMIP6 ScenarioMIP ssp245. Version 20200512.* Earth System Grid Federation. doi: 10.22033/ESGF/CMIP6.9209

Song, Z., Qiao, F., Bao, Y., Shu, Q., Song, Y., & Yang, X. (2019d). *FIO-QLNM FIO-ESM2.0*

model output prepared for CMIP6 ScenarioMIP ssp585. Version 20200512. Earth System Grid Federation. doi: 10.22033/ESGF/CMIP6.9214

Swart, N. C., Cole, J. N., Kharin, V. V., Lazare, M., Scinocca, J. F., Gillett, N. P., . . . Sigmond, M. (2019a). *CCCma CanESM5-CanOE model output prepared for CMIP6 CMIP historical. Version 20200512.* Earth System Grid Federation. doi: 10.22033/ESGF/CMIP6.10260

Swart, N. C., Cole, J. N., Kharin, V. V., Lazare, M., Scinocca, J. F., Gillett, N. P., . . . Sigmond, M. (2019b). *CCCma CanESM5-CanOE model output prepared for CMIP6 ScenarioMIP ssp126. Version 20200512.* Earth System Grid Federation. doi: 10.22033/ESGF/CMIP6.10269

Swart, N. C., Cole, J. N., Kharin, V. V., Lazare, M., Scinocca, J. F., Gillett, N. P., . . . Sigmond, M. (2019c). *CCCma CanESM5-CanOE model output prepared for CMIP6 ScenarioMIP ssp245. Version 20200512.* Earth System Grid Federation. doi: 10.22033/ESGF/CMIP6.10270

Swart, N. C., Cole, J. N., Kharin, V. V., Lazare, M., Scinocca, J. F., Gillett, N. P., . . . Sigmond, M. (2019d). *CCCma CanESM5-CanOE model output prepared for CMIP6 ScenarioMIP ssp370. Version 20200512.* Earth System Grid Federation. doi: 10.22033/ESGF/CMIP6.10271

Swart, N. C., Cole, J. N., Kharin, V. V., Lazare, M., Scinocca, J. F., Gillett, N. P., . . . Sigmond, M. (2019e). *CCCma CanESM5-CanOE model output prepared for CMIP6 ScenarioMIP ssp585. Version 20200512.* Earth System Grid Federation. doi: 10.22033/ESGF/CMIP6.10276

Swart, N. C., Cole, J. N., Kharin, V. V., Lazare, M., Scinocca, J. F., Gillett, N. P., . . . Sigmond,

M. (2019f). *CCCma CanESM5 model output prepared for CMIP6 CMIP historical. Version 20200512.* Earth System Grid Federation. doi: 10.22033/ESGF/CMIP6.3610

Swart, N. C., Cole, J. N., Kharin, V. V., Lazare, M., Scinocca, J. F., Gillett, N. P., ... Sigmond, M. (2019g). *CCCma CanESM5 model output prepared for CMIP6 ScenarioMIP ssp126. Version 20200512.* Earth System Grid Federation. doi: 10.22033/ESGF/CMIP6.3683

Swart, N. C., Cole, J. N., Kharin, V. V., Lazare, M., Scinocca, J. F., Gillett, N. P., ... Sigmond, M. (2019h). *CCCma CanESM5 model output prepared for CMIP6 ScenarioMIP ssp245. Version 20200512.* Earth System Grid Federation. doi: 10.22033/ESGF/CMIP6.3685

Swart, N. C., Cole, J. N., Kharin, V. V., Lazare, M., Scinocca, J. F., Gillett, N. P., ... Sigmond, M. (2019i). *CCCma CanESM5 model output prepared for CMIP6 ScenarioMIP ssp370. Version 20200512.* Earth System Grid Federation. doi: 10.22033/ESGF/CMIP6.3690

Swart, N. C., Cole, J. N., Kharin, V. V., Lazare, M., Scinocca, J. F., Gillett, N. P., ... Sigmond, M. (2019j). *CCCma CanESM5 model output prepared for CMIP6 ScenarioMIP ssp585. Version 20200512.* Earth System Grid Federation. doi: 10.22033/ESGF/CMIP6.3696

Tachiiri, K., Abe, M., Hajima, T., Arakawa, O., Suzuki, T., Komuro, Y., ... Kawamiya, M. (2019a). *MIROC MIROC-ES2L model output prepared for CMIP6 ScenarioMIP ssp126. Version 20200512.* Earth System Grid Federation. doi: 10.22033/ESGF/CMIP6.5742

Tachiiri, K., Abe, M., Hajima, T., Arakawa, O., Suzuki, T., Komuro, Y., ... Kawamiya, M. (2019b). *MIROC MIROC-ES2L model output prepared for CMIP6 ScenarioMIP ssp245. Version 20200512.* Earth System Grid Federation. doi: 10.22033/ESGF/CMIP6.5745

Tachiiri, K., Abe, M., Hajima, T., Arakawa, O., Suzuki, T., Komuro, Y., ... Kawamiya, M. (2019c). *MIROC MIROC-ES2L model output prepared for CMIP6 ScenarioMIP ssp370.*

Version 20200512. Earth System Grid Federation. doi: 10.22033/ESGF/CMIP6.5751

Tachiiri, K., Abe, M., Hajima, T., Arakawa, O., Suzuki, T., Komuro, Y., ... Kawamiya, M. (2019d). *MIROC MIROC-ES2L model output prepared for CMIP6 ScenarioMIP ssp585. Version 20200512.* Earth System Grid Federation. doi: 10.22033/ESGF/CMIP6.5770

Tang, Y., Rumbold, S., Ellis, R., Kelley, D., Mulcahy, J., Sellar, A., ... Jones, C. (2019). *MOHC UKESM1.0-LL model output prepared for CMIP6 CMIP historical. Version 20200512.* Earth System Grid Federation. doi: 10.22033/ESGF/CMIP6.6113

Tatebe, H., & Watanabe, M. (2018). *MIROC MIROC6 model output prepared for CMIP6 CMIP historical. Version 20200512.* Earth System Grid Federation. doi: 10.22033/ESGF/CMIP6.5603

Voldoire, A. (2018). *CMIP6 simulations of the CNRM-CERFACS based on CNRM-CM6-1 model for CMIP experiment historical. Version 20200512.* Earth System Grid Federation. doi: 10.22033/ESGF/CMIP6.4066

Voldoire, A. (2019a). *CNRM-CERFACS CNRM-CM6-1-HR model output prepared for CMIP6 CMIP historical. Version 20200512.* Earth System Grid Federation. doi: 10.22033/ESGF/CMIP6.4067

Voldoire, A. (2019b). *CNRM-CERFACS CNRM-CM6-1-HR model output prepared for CMIP6 ScenarioMIP ssp245. Version 20200512.* Earth System Grid Federation. doi: 10.22033/ESGF/CMIP6.4190

Voldoire, A. (2019c). *CNRM-CERFACS CNRM-CM6-1-HR model output prepared for CMIP6 ScenarioMIP ssp585. Version 20200512.* Earth System Grid Federation. doi: 10.22033/ESGF/CMIP6.4225

Voldoire, A. (2019d). *CNRM-CERFACS CNRM-CM6-1 model output prepared for CMIP6 ScenarioMIP ssp126. Version 20200512.* Earth System Grid Federation. doi: 10.22033/ESGF/CMIP6.4184

Voldoire, A. (2019e). *CNRM-CERFACS CNRM-CM6-1 model output prepared for CMIP6 ScenarioMIP ssp245. Version 20200512.* Earth System Grid Federation. doi: 10.22033/ESGF/CMIP6.4189

Voldoire, A. (2019f). *CNRM-CERFACS CNRM-CM6-1 model output prepared for CMIP6 ScenarioMIP ssp370. Version 20200512.* Earth System Grid Federation. doi: 10.22033/ESGF/CMIP6.4197

Voldoire, A. (2019g). *CNRM-CERFACS CNRM-CM6-1 model output prepared for CMIP6 ScenarioMIP ssp585. Version 20200512.* Earth System Grid Federation. doi: 10.22033/ESGF/CMIP6.4224

Voldoire, A. (2019h). *CNRM-CERFACS CNRM-ESM2-1 model output prepared for CMIP6 ScenarioMIP ssp126. Version 20200512.* Earth System Grid Federation. doi: 10.22033/ESGF/CMIP6.4186

Voldoire, A. (2019i). *CNRM-CERFACS CNRM-ESM2-1 model output prepared for CMIP6 ScenarioMIP ssp245. Version 20200512.* Earth System Grid Federation. doi: 10.22033/ESGF/CMIP6.4191

Voldoire, A. (2019j). *CNRM-CERFACS CNRM-ESM2-1 model output prepared for CMIP6 ScenarioMIP ssp370. Version 20200512.* Earth System Grid Federation. doi: 10.22033/ESGF/CMIP6.4199

Voldoire, A. (2019k). *CNRM-CERFACS CNRM-ESM2-1 model output prepared for CMIP6*

ScenarioMIP ssp585. Version 20200512. Earth System Grid Federation. doi: 10.22033/ESGF/CMIP6.4226

Voldoire, A. (2020a). *CNRM-CERFACS CNRM-CM6-1-HR model output prepared for CMIP6 ScenarioMIP ssp126. Version 20200512.* Earth System Grid Federation. doi: 10.22033/ESGF/CMIP6.4185

Voldoire, A. (2020b). *CNRM-CERFACS CNRM-CM6-1-HR model output prepared for CMIP6 ScenarioMIP ssp370. Version 20200512.* Earth System Grid Federation. doi: 10.22033/ESGF/CMIP6.4198

Volodin, E., Mortikov, E., Gritsun, A., Lykossov, V., Galin, V., Diansky, N., ... Emelina, S. (2019a). *INM INM-CM4-8 model output prepared for CMIP6 CMIP historical. Version 20200512.* Earth System Grid Federation. doi: 10.22033/ESGF/CMIP6.5069

Volodin, E., Mortikov, E., Gritsun, A., Lykossov, V., Galin, V., Diansky, N., ... Emelina, S. (2019b). *INM INM-CM4-8 model output prepared for CMIP6 ScenarioMIP ssp126. Version 20200512.* Earth System Grid Federation. doi: 10.22033/ESGF/CMIP6.12325

Volodin, E., Mortikov, E., Gritsun, A., Lykossov, V., Galin, V., Diansky, N., ... Emelina, S. (2019c). *INM INM-CM4-8 model output prepared for CMIP6 ScenarioMIP ssp245. Version 20200512.* Earth System Grid Federation. doi: 10.22033/ESGF/CMIP6.12327

Volodin, E., Mortikov, E., Gritsun, A., Lykossov, V., Galin, V., Diansky, N., ... Emelina, S. (2019d). *INM INM-CM4-8 model output prepared for CMIP6 ScenarioMIP ssp370. Version 20200512.* Earth System Grid Federation. doi: 10.22033/ESGF/CMIP6.12329

Volodin, E., Mortikov, E., Gritsun, A., Lykossov, V., Galin, V., Diansky, N., ... Emelina, S. (2019e). *INM INM-CM4-8 model output prepared for CMIP6 ScenarioMIP ssp585. Version*

20200512. Earth System Grid Federation. doi: 10.22033/ESGF/CMIP6.12337

Volodin, E., Mortikov, E., Gritsun, A., Lykossov, V., Galin, V., Diansky, N., ... Emelina, S. (2019f). *INM INM-CM5-0 model output prepared for CMIP6 CMIP historical. Version 20200512.* Earth System Grid Federation. doi: 10.22033/ESGF/CMIP6.5070

Volodin, E., Mortikov, E., Gritsun, A., Lykossov, V., Galin, V., Diansky, N., ... Emelina, S. (2019g). *INM INM-CM5-0 model output prepared for CMIP6 ScenarioMIP ssp126. Version 20200512.* Earth System Grid Federation. doi: 10.22033/ESGF/CMIP6.12326

Volodin, E., Mortikov, E., Gritsun, A., Lykossov, V., Galin, V., Diansky, N., ... Emelina, S. (2019h). *INM INM-CM5-0 model output prepared for CMIP6 ScenarioMIP ssp245. Version 20200512.* Earth System Grid Federation. doi: 10.22033/ESGF/CMIP6.12328

Volodin, E., Mortikov, E., Gritsun, A., Lykossov, V., Galin, V., Diansky, N., ... Emelina, S. (2019i). *INM INM-CM5-0 model output prepared for CMIP6 ScenarioMIP ssp370. Version 20200512.* Earth System Grid Federation. doi: 10.22033/ESGF/CMIP6.12330

Volodin, E., Mortikov, E., Gritsun, A., Lykossov, V., Galin, V., Diansky, N., ... Emelina, S. (2019j). *INM INM-CM5-0 model output prepared for CMIP6 ScenarioMIP ssp585. Version 20200512.* Earth System Grid Federation. doi: 10.22033/ESGF/CMIP6.12338

Wieners, K.-H., Giorgetta, M., Jungclaus, J., Reick, C., Esch, M., Bittner, M., ... Roeckner, E. (2019a). *MPI-M MPI-ESM1.2-LR model output prepared for CMIP6 CMIP historical. Version 20200512.* Earth System Grid Federation. doi: 10.22033/ESGF/CMIP6.6595

Wieners, K.-H., Giorgetta, M., Jungclaus, J., Reick, C., Esch, M., Bittner, M., ... Roeckner, E. (2019b). *MPI-M MPI-ESM1.2-LR model output prepared for CMIP6 ScenarioMIP ssp126. Version 20200512.* Earth System Grid Federation. doi: 10.22033/ESGF/CMIP6.6690

Wieners, K.-H., Giorgetta, M., Jungclaus, J., Reick, C., Esch, M., Bittner, M., ... Roeckner, E. (2019c). *MPI-M MPI-ESM1.2-LR model output prepared for CMIP6 ScenarioMIP ssp245. Version 20200512.* Earth System Grid Federation. doi: 10.22033/ESGF/CMIP6.6693

Wieners, K.-H., Giorgetta, M., Jungclaus, J., Reick, C., Esch, M., Bittner, M., ... Roeckner, E. (2019d). *MPI-M MPI-ESM1.2-LR model output prepared for CMIP6 ScenarioMIP ssp370. Version 20200512.* Earth System Grid Federation. doi: 10.22033/ESGF/CMIP6.6695

Wieners, K.-H., Giorgetta, M., Jungclaus, J., Reick, C., Esch, M., Bittner, M., ... Roeckner, E. (2019e). *MPI-M MPI-ESM1.2-LR model output prepared for CMIP6 ScenarioMIP ssp585. Version 20200512.* Earth System Grid Federation. doi: 10.22033/ESGF/CMIP6.6705

Wu, T., Chu, M., Dong, M., Fang, Y., Jie, W., Li, J., ... Zhang, Y. (2018). *BCC BCC-CSM2MR model output prepared for CMIP6 CMIP historical. Version 20200512.* Earth System Grid Federation. doi: 10.22033/ESGF/CMIP6.2948

Xin, X., Wu, T., Shi, X., Zhang, F., Li, J., Chu, M., ... Wei, M. (2019a). *BCC BCC-CSM2MR model output prepared for CMIP6 ScenarioMIP ssp126. Version 20200512.* Earth System Grid Federation. doi: 10.22033/ESGF/CMIP6.3028

Xin, X., Wu, T., Shi, X., Zhang, F., Li, J., Chu, M., ... Wei, M. (2019b). *BCC BCC-CSM2MR model output prepared for CMIP6 ScenarioMIP ssp245. Version 20200512.* Earth System Grid Federation. doi: 10.22033/ESGF/CMIP6.3030

Xin, X., Wu, T., Shi, X., Zhang, F., Li, J., Chu, M., ... Wei, M. (2019c). *BCC BCC-CSM2MR model output prepared for CMIP6 ScenarioMIP ssp370. Version 20200512.* Earth System Grid Federation. doi: 10.22033/ESGF/CMIP6.3035

Xin, X., Wu, T., Shi, X., Zhang, F., Li, J., Chu, M., ... Wei, M. (2019d). *BCC BCC-*

CSM2MR model output prepared for CMIP6 ScenarioMIP ssp585. Version 20200512. Earth System Grid Federation. Retrieved from https://doi.org/10.22033/ESGF/CMIP6.3050 doi: 10.22033/ESGF/CMIP6.3050

YU, Y. (2019a). *CAS FGOALS-f3-L model output prepared for CMIP6 CMIP historical. Version 20200512.* Earth System Grid Federation. doi: 10.22033/ESGF/CMIP6.3355

YU, Y. (2019b). *CAS FGOALS-f3-L model output prepared for CMIP6 ScenarioMIP ssp126. Version 20200512.* Earth System Grid Federation. doi: 10.22033/ESGF/CMIP6.3464

YU, Y. (2019c). *CAS FGOALS-f3-L model output prepared for CMIP6 ScenarioMIP ssp245. Version 20200512.* Earth System Grid Federation. doi: 10.22033/ESGF/CMIP6.3468

YU, Y. (2019d). *CAS FGOALS-f3-L model output prepared for CMIP6 ScenarioMIP ssp370. Version 20200512.* Earth System Grid Federation. doi: 10.22033/ESGF/CMIP6.3479

YU, Y. (2019e). *CAS FGOALS-f3-L model output prepared for CMIP6 ScenarioMIP ssp585. Version 20200512.* Earth System Grid Federation. doi: 10.22033/ESGF/CMIP6.3502

Yukimoto, S., Koshiro, T., Kawai, H., Oshima, N., Yoshida, K., Urakawa, S., … Adachi, Y. (2019a). *MRI MRI-ESM2.0 model output prepared for CMIP6 CMIP historical. Version 20200512.* Earth System Grid Federation. doi: 10.22033/ESGF/CMIP6.6842

Yukimoto, S., Koshiro, T., Kawai, H., Oshima, N., Yoshida, K., Urakawa, S., … Adachi, Y. (2019b). *MRI MRI-ESM2.0 model output prepared for CMIP6 ScenarioMIP ssp126. Version 20200512.* Earth System Grid Federation. doi: 10.22033/ESGF/CMIP6.6909

Yukimoto, S., Koshiro, T., Kawai, H., Oshima, N., Yoshida, K., Urakawa, S., … Adachi, Y. (2019c). *MRI MRI-ESM2.0 model output prepared for CMIP6 ScenarioMIP ssp245. Version 20200512.* Earth System Grid Federation. doi: 10.22033/ESGF/CMIP6.6910

Yukimoto, S., Koshiro, T., Kawai, H., Oshima, N., Yoshida, K., Urakawa, S., … Adachi, Y. (2019d). *MRI MRI-ESM2.0 model output prepared for CMIP6 ScenarioMIP ssp370. Version 20200512.* Earth System Grid Federation. doi: 10.22033/ESGF/CMIP6.6915

Yukimoto, S., Koshiro, T., Kawai, H., Oshima, N., Yoshida, K., Urakawa, S., … Adachi, Y. (2019e). *MRI MRI-ESM2.0 model output prepared for CMIP6 ScenarioMIP ssp585. Version 20200512.* Earth System Grid Federation. doi: 10.22033/ESGF/CMIP6.6929

Zhang, J., Wu, T., Shi, X., Zhang, F., Li, J., Chu, M., … Wei, M. (2018). *BCC BCC-ESM1 model output prepared for CMIP6 CMIP historical. Version 20200512.* Earth System Grid Federation. doi: 10.22033/ESGF/CMIP6.2949

Ziehn, T., Chamberlain, M., Lenton, A., Law, R., Bodman, R., Dix, M., … Druken, K. (2019a). *CSIRO ACCESS-ESM1.5 model output prepared for CMIP6 CMIP historical. Version 20200512.* Earth System Grid Federation. doi: 10.22033/ESGF/CMIP6.4272

Ziehn, T., Chamberlain, M., Lenton, A., Law, R., Bodman, R., Dix, M., … Druken, K. (2019b). *CSIRO ACCESS-ESM1.5 model output prepared for CMIP6 ScenarioMIP ssp126. Version 20200512.* Earth System Grid Federation. doi: 10.22033/ESGF/CMIP6.4320

Ziehn, T., Chamberlain, M., Lenton, A., Law, R., Bodman, R., Dix, M., … Druken, K. (2019c). *CSIRO ACCESS-ESM1.5 model output prepared for CMIP6 ScenarioMIP ssp245. Version 20200512.* Earth System Grid Federation. doi: 10.22033/ESGF/CMIP6.4322

Ziehn, T., Chamberlain, M., Lenton, A., Law, R., Bodman, R., Dix, M., … Druken, K. (2019d). *CSIRO ACCESS-ESM1.5 model output prepared for CMIP6 ScenarioMIP ssp370. Version 20200512.* Earth System Grid Federation. doi: 10.22033/ESGF/CMIP6.4324

Ziehn, T., Chamberlain, M., Lenton, A., Law, R., Bodman, R., Dix, M., … Druken, K. (2019e).

CSIRO ACCESS-ESM1.5 model output prepared for CMIP6 ScenarioMIP ssp585. Version 20200512. Earth System Grid Federation. doi: 10.22033/ESGF/CMIP6.4333
